# A Novel Method for Creating Heterologous Lethal Antibiotic Producers by Screening from Combi-OGAB Library with Various Promoters in a Biosynthetic Gene Cluster

**DOI:** 10.1021/acsomega.3c08240

**Published:** 2024-01-30

**Authors:** Naoki Miyamoto, Akinori Nishigami, Nao Hosoda, Kentaro Hayashi, Naoyuki Yamada, Kenji Tsuge

**Affiliations:** Synplogen Co., Ltd., Kobe, Hyogo 6500047, Japan

## Abstract

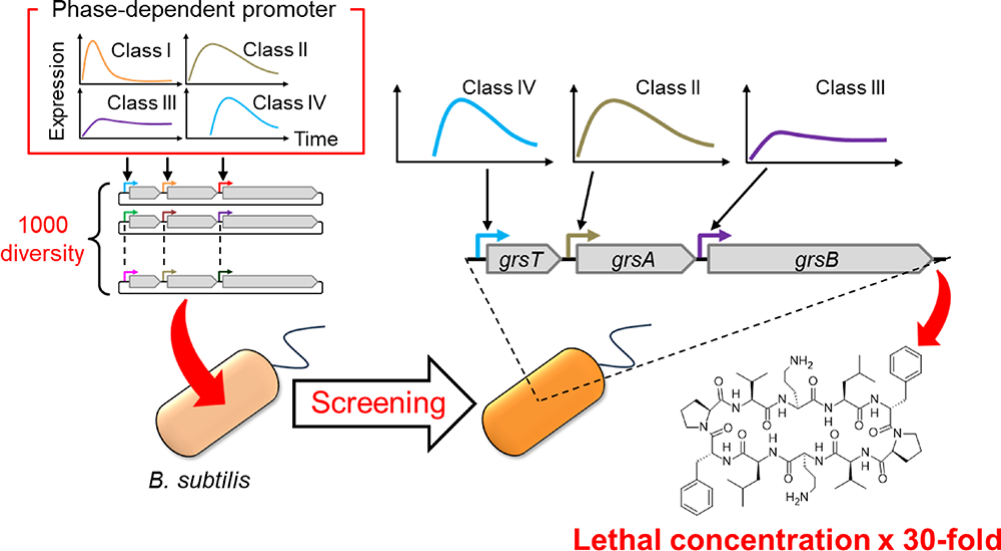

In this study, we devised a novel method to create heterologous producers of lethal antibiotics against host bacteria. Heterologous producers cannot be created when antibiotics are toxic to host bacteria. To overcome this challenge, we developed a novel method involving construction of a combinatorial library with various promoters and screening based on the production. To realize this, we utilized Combi-OGAB (**Combi**natorial **O**rdered **G**ene **A**ssembly in *Bacillus subtilis*), which technology can effectively construct diverse combinatorial library and accelerate screening procedures. *B. subtilis* and Gramicidin S were selected as the host bacterium and the targeted antibiotic, respectively. The screened producer from Combi-OGAB screening cycles achieved >30-fold productivity over the lethal level. These results suggest that our strategy has the potential to maximize the phenotypic resistance of host bacteria to create heterologous lethal antibiotic producers.

## Introduction

Discovery of novel antibiotics is necessary to overcome antimicrobial resistance, which is one of the most urgent global threats to human health.^1^ Bacteria are among the most proficient producers of antibiotics. Genome mining has revealed the presence of novel biosynthetic gene clusters (BGCs) for antibiotics within bacterial genomes.^1^ From BGCs, product types such as peptides, polyketides, or terpenes can be estimated; however, it is difficult to link between BGCs, chemical structures, and bioactivities of produced antibiotics.^2^ It is estimated that 99% or more of all bacteria are uncultivable,^3^ requiring the use of heterologous hosts for their production. When host bacteria do not possess resistance against the produced antibiotics, they cannot survive and valuable antibiotics cannot be found. In this study, we propose a novel method for generating heterologous antibiotic producers with transient resistance (phenotypic resistance)^4^ by fine-tuning the timing of bioproduction using *Bacillus subtilis* and Gramicidin Soviet (Gramicidin S; GS) as the host bacteria and targeted antibiotic, respectively.

**Figure 1:**
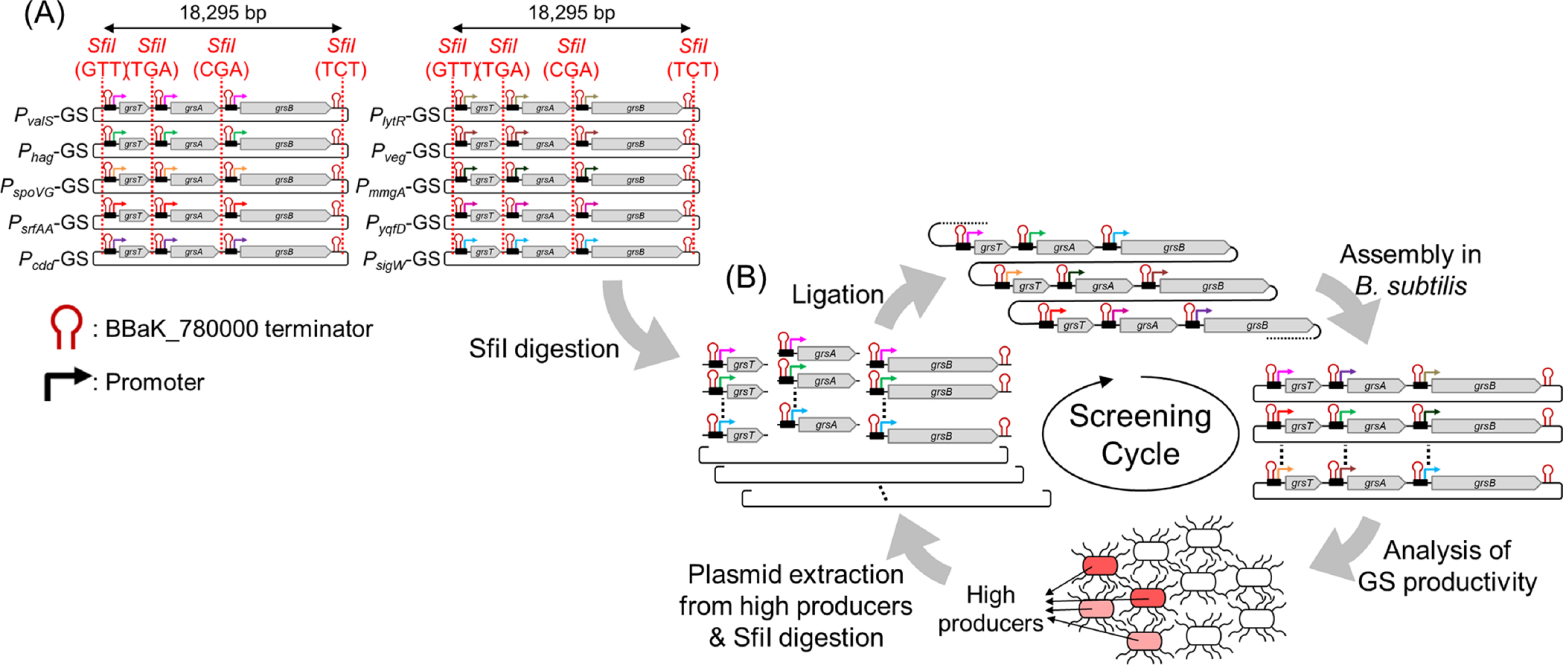
Schematic image of the Combi-OGAB screening procedure in this study. (A) Ten plasmids were prepared for the initial library construction. Each plasmid has one kind of promoter of *B. subtilis* for all three genes. (B) Combi-OGAB screening procedure. The constructed plasmids were equimolarly mixed, and they were digested with SfiI. Digested DNA fragments were ligated to tandem-repeat form to shuffle promoter sequences in accordance with sticky ends generated by SfiI. *B. subtilis* was transformed with this ligated DNA, and library plasmids with shuffled promoters were generated in each *B. subtilis* clone. GS productivity of each clone was analyzed, and high producers among analyzed clones were selected. Their plasmid mixture was digested with SfiI for the construction of the second screening cycle. These procedures were repeated until productivity was enriched.

GS is a non-ribosomal peptide (NRP)^5^ with antimicrobial activity against gram-positive bacteria,^6^ especially *B. subtilis*. Its molecular structure comprises a head-to-tail dimer of ^D^Phe-^L^Pro-^L^Val-^L^Orn-^L^Leu. The GS BGC comprises three genes: *grsT* (encoding thioesterase GrsT), *grsA* (encoding ^L^Phe isomerase GrsA), and *grsB* (encoding core peptide synthetase GrsB). In *Aneuribacillus migulanus*, a single promoter *P*_*grs*_ polycistronically transcribes all three genes.^7^ However, these genes are not associated with GS resistance. The positively charged residues of GS account for its initial binding to the cell membrane, and the hydrophobic domain promotes its further insertion into the lipid layer, destroying the cell membrane.^8^ The native producer, *A. migulanus*, is a gram-positive bacterium that accumulates GS in granules inside the cell, serving as a self-protection system.^9^ In contrast, *B. subtilis*, a gram-positive bacterium, is effectively killed by GS (minimum inhibitory concentration [MIC] = 1.7 μM [= 1.9 μg/mL]).^10^*B. subtilis* is considered a potential host bacterium for heterologous NRP producers, as it natively produces some NRPs, such as surfactin,^11^ iturin,^12^ and plipastatin.^13^ In addition, *B. subtilis* is a common host bacterium for genetic modifications; it is utilized in the final step to construct plasmid DNA for technologies of gene synthesis, called OGAB (**O**rdered **G**ene **A**ssembly in *Bacillus subtilis*)^14,15^ and combinatorial library construction called Combi-OGAB (**Combi**natorial **OGAB**; procedures are depicted in Figure 1).

Fine-tuning the balance between the growth and production of producers is necessary to enhance the productivity of metabolites.^16,17^ Phenotypic resistance is associated with specific processes, such as growth in biofilms, the stationary growth phase, or persistence.^4^ Some promoters initiate gene transcription at different growth phases in the host bacteria.^18−20^ Therefore, we hypothesized that growth phase-dependent promoters would enable the optimization of bioproduction toward a suitable growth phase for the host bacterium and that the timing of antibiotic production could be controlled to the high phenotypic resistance phase to enhance productivity.

In the previous study, they monitored time profiles of eGFP expression driven by 114 *B. subtilis* promoters and classified those promoters as “growth phase-dependent promoters” in four classes (Class I: exponential phase, Class II: middle-log and early stationary phase, Class III: lag-log and stationary phase, and Class IV: stationary phase).^18^ We hypothesized that polycistronic transcription of *grs* genes could be suitable for GS production in *A. migulanus*, but it is unclear whether this is also the case in heterologous hosts. It is possible that each gene has a suitable transcription phase to maximize the productivity in heterologous hosts. Therefore, we designed monocistronic transcription in GS BGC, aiming to establish the creation of heterologous GS producers of *B. subtilis* by optimizing the suitable transcription phase and strength for each gene. After constructing a plasmid library for GS production in *B. subtilis*, we directly assessed the GS productivity.

## Results

### Promoter Selection for Library Construction

In this study, we selected 10 promoters (Class I: *P*_*valS*_ and *P*_*hag*_, Class II: *P*_*spoVG*_, *P*_*srfAA*_, and *P*_*cdd*_, Class III: *P*_*lytR*_ and *P*_*veg*_, and Class IV: *P*_*mmgA*_, *P*_*yqfD*_, and *P*_*sigW*_) with different transcription phases and transcription strengths in each class. As described above, three genes, *grsT*, *grsA*, and *grsB*, comprise an operon in the native producer,^7^ so we targeted all three genes for fine-tuning their transcription. The promoters were integrated into a combinatorial promoter library to screen suitable promoters for *grsT*, *grsA*, and *grsB* to convert *B. subtilis* to the GS producer.

### Preparation of Plasmids for Library Construction

**Figure 2:**
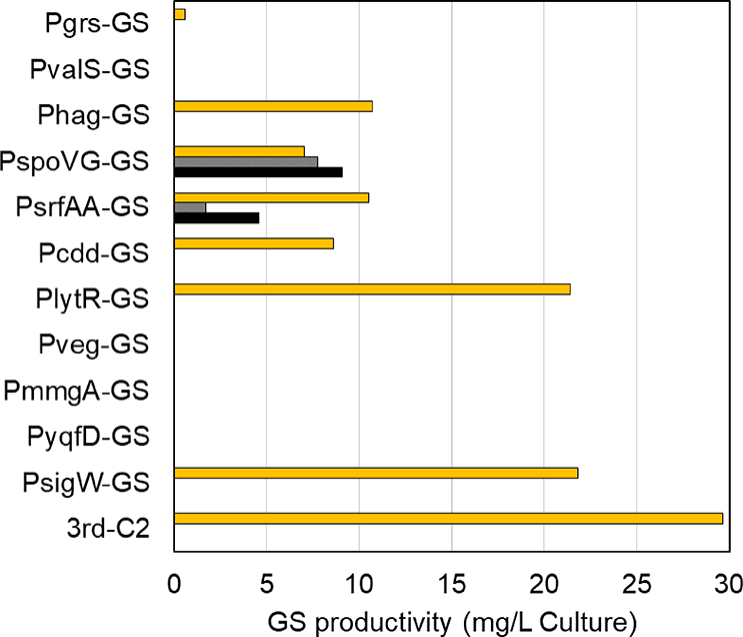
GS productivity was driven by the native BGC state in *A. migulanus* (*P*_*grs*_-GS), prepared plasmids for initial library construction (*P*_*valS*_-GS, *P*_*hag*_-GS, *P*_*spoVG*_-GS, *P*_*srfAA*_-GS, *P*_*cdd*_-GS, *P*_*lytR*_-GS, *P*_*veg*_-GS, *P*_*mmgA*_-GS, *P*_*yqfD*_-GS, and *P*_*sigW*_-GS), and the screened highest promoter set (3rd-C2). Yellow, gray, and black bars indicate the productivity of GS, [Lys-4]-GS, and [Lys-4, 9]-GS detected via HPLC, respectively. Compared with the GS productivity of the native BGC state (*P*_*grs*_-GS, about 0.6 mg/L), *P*_*sigW*_-GS and 3rd-C2 showed 37.8-fold and 51.4-fold higher productivity, respectively. Moreover, 3rd-C2 showed 1.36-fold higher productivity than *P*_*sigW*_-GS.

First, we constructed 10 GS BGC plasmids with monocistronic transcription (Figure 1A). The GS productivity driven by these 10 promoters was analyzed. Compared with the native transcription state (*P*_*grs*_-GS: *P*_*grs*_-*grsT*-*grsA*-*grsB*, GS productivity = approximately 0.6 mg/L culture), some of them showed higher productivity than the native BGC state (Figure 2). In particular, *P*_*sigW*_-GS resulted in approximately 37.8-fold higher GS productivity (approximately 21.8 mg/L of culture) than *P*_*grs*_-GS (Figure 2). *P*_*spoVG*_-GS and *P*_*srfAA*_-GS showed three peaks on their high-performance liquid chromatography (HPLC) chromatograms in the GS fraction, suggesting potential byproduct formation. Liquid chromatography–mass spectrometry (LC–MS) analysis of the extracted samples revealed three *m*/*z* values: 1141 (GS), 1155, and 1169 (Figure S1).

In a previous report, the ^L^Orn selective module in GrsB possessed substrate specificity to ^L^Lys as well;^21^ thus, ^L^Lys-substituted GS molecules ([Lys-4]-GS and [Lys-4, 9]-GS) were detected as byproducts in the GS fraction of native producers.^22^ Moreover, [Lys-4, 9]-GS showed lower antibiotic activity than GS (approximately half).^23^ In our study, analysis of GS productivity revealed that *P*_*spoVG*_ and *P*_*srfAA*_ might have the potential to produce byproducts; however, it was unclear whether the transcription of *grsT*, *grsA*, and/or *grsB* by these promoters is critical for byproduct production. If *P*_*spoVG*_-GS and *P*_*srfAA*_-GS were excluded from the combinatorial library, then the initial diversity would have been reduced. Therefore, *P*_*spoVG*_-GS and *P*_*srfAA*_-GS were integrated into the initial library.

### Combi-OGAB Screening to Gain GS Producing *B. subtilis*

After the initial library construction, Combi-OGAB screening cycles were conducted (Figure 1B). Equimolarly mixed plasmids were digested with SfiI, and the digested samples were ligated to the tandem-repeat form. Sticky ends generated by SfiI defined the ligation order; therefore, the correct BGC units with shuffled promoters were included in the tandem-repeat DNA. Then, *B. subtilis* was transformed with that tandem-repeat DNA, and plasmids with a combinatorial promoter library were assembled in *B. subitilis* (theoretical diversity: 1,000).^15^ This assembly host *B. subtilis* holds pUB8, which contains *lpa-8* coding 4′-phosphopantetheinyl transferase to activate the production mechanism of NRP.^13^ Therefore, the assembly hosts were directly transferred to the production hosts for the GS productivity analysis.

**Figure 3:**
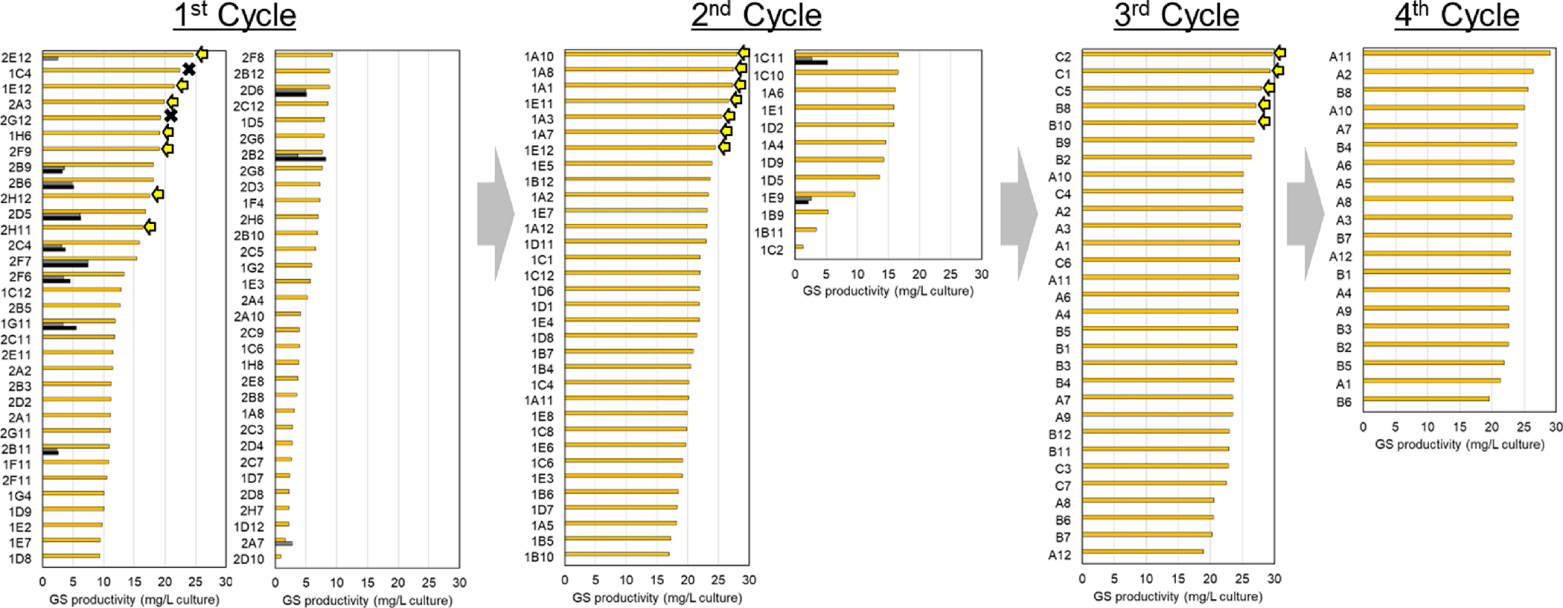
GS and byproduct productivity of producers in four cycles. Yellow, gray, and black bars indicate the productivity of GS, [Lys-4]-GS, and [Lys-4, 9]-GS detected via HPLC, respectively. Yellow arrows indicate selected clones for the next cycle. X-marked clones could not grow again after the production experiment, and their plasmids for the next cycle could not be collected. The highest producer, 3rd-C2, achieved 29 ± 0.73 mg/L culture (*N* = 3) of GS productivity.

**Figure 4:**
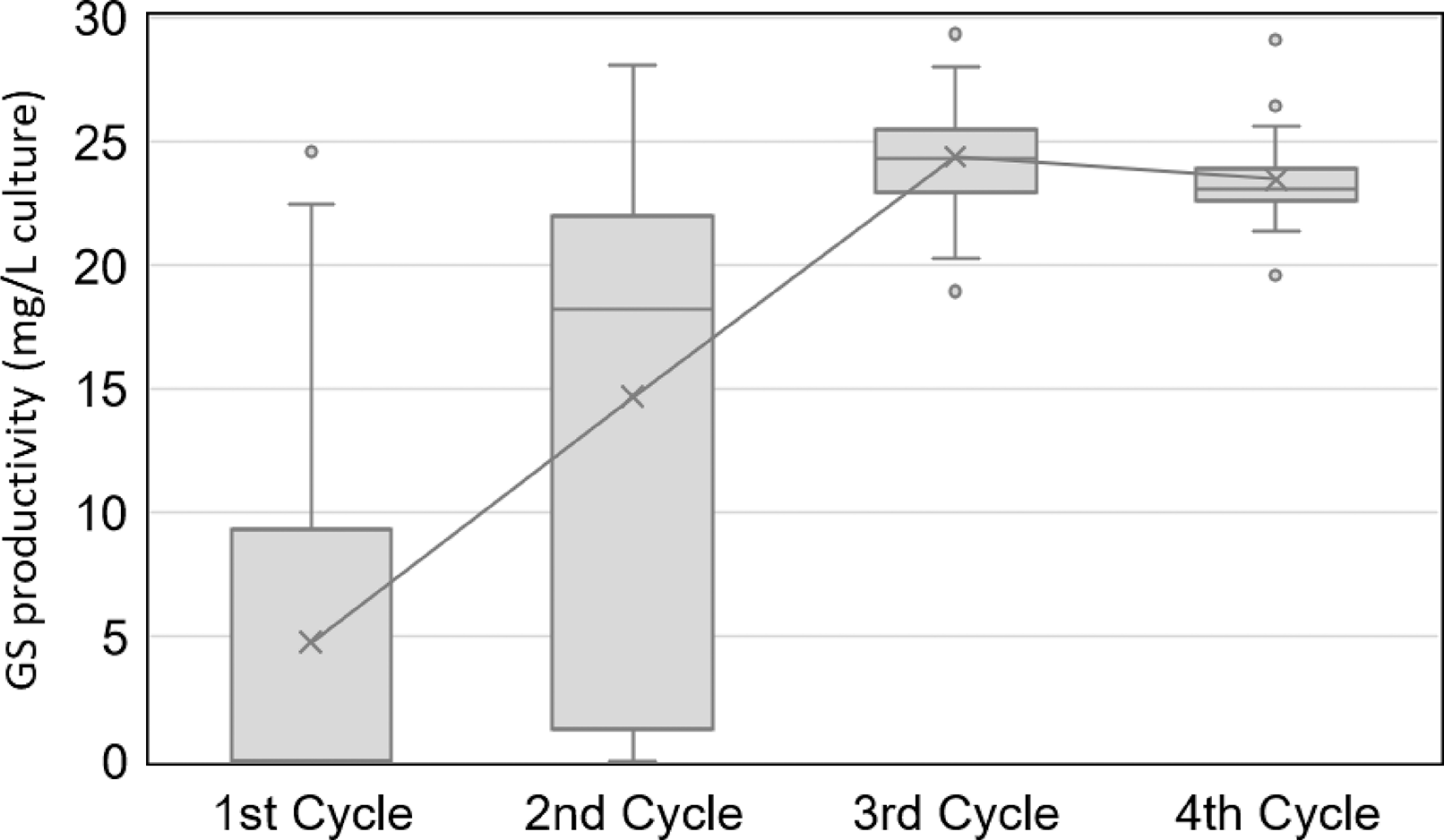
GS productivity transition among the four cycles. The productivity was enriched in the 3rd cycle. The 1st, 2nd, 3rd, and 4th cycles include 192, 60, 30, and 20 clones, respectively.

Next, the GS productivity of the transformants was analyzed individually (Table S1 and Figure 3). In total, 192 transformants from more than 10,000 transformants were cultured to produce GS, and 134 clones were grown. Among these, GS was detected in 65 clones via HPLC, and 69 clones were non-producers. The GS productivity of 65 producers varied from 0.86 to 24.6 mg/L of culture (Figures 3 and 4). Some of these clones exhibited byproduct production. The byproducts exhibit decreased antibiotic activity as described above;^23^ therefore, we selected producers with high GS production against the byproducts for the second cycle.

The top nine GS-producing clones were cultured to collect plasmids for the second cycle-library construction, and two clones (1C4 and 2G12) were not grown. Therefore, seven clones were used for the second library construction. Equal volumes of the cultures were mixed, and plasmids were collected from this mixture. This construction procedure is different from that of the first library, described above. The plasmid mixture was digested with SfiI again and ligated to construct the second library in the same way as that in the 1^st^ library construction. Sixty clones from over 10,000 transformants were selected and cultured, of which 59 were viable. Of these 59 clones, 45 were producers. Except for 2 clones, 43 clones showed a single GS peak on HPLC. The top seven producers were selected for the construction of the third library in the same way as in the second cycle. In the third screening cycle, 30 clones from over 10,000 transformants were selected, all of which were viable GS producers with undetectable byproducts. The top five producers were transferred to the fourth cycle, and all 20 clones from over 10,000 transformants were culturable GS producers.

Through four screening cycles, the GS productivity was enriched at the third cycle (Figure 4). Among the four cycles, the third-cycle C2 clone (3rd-C2) showed the highest GS productivity (29 ± 0.73 mg/L culture, *N* = 3), which is approximately 50-fold and 1.4-fold higher than *P*_*grs*_-GS and *P*_*sigW*_-GS, respectively (Figure 1). Next, we examined the exogenous GS MIC of 3rd-C2, which was <1 μg/mL (Figure S2), which is consistent with the reported value for *B. subtilis* (1.9 μg/mL).^10^ Thus, the GS sensitivity of 3rd-C2 was retained, indicating that 3rd-C2 producer exhibits phenotypic resistance without genetic alteration against GS.^4^ These results demonstrate that we successfully optimized monocistronic promoters in GS BGC and created a GS producer with productivity over 30-fold the lethal level.

### Properties of Screened GS Producer 3rd-C2

In the GS fraction produced by 3rd-C2, GS, [Lys-4]-GS, and [Lys-4, 9]-GS ratios were 95.56%, 3.58%, and 0.86%, respectively, as determined using LC-MS. DNA sequencing analysis revealed that 3rd-C2 holds *P*_*sigW*_-*grsT*-*P*_*cdd*_-*grsA*-*P*_*lytR*_-*grsB*. The transcription phases of *P*_*sigW*_, *P*_*cdd*_, and *P*_*lytR*_ are the stationary phase (Class IV), middle-log and early stationary phase (Class II), and lag-log and stationary phase (Class III), respectively, and their transcriptional strength is not necessarily strong in each class.^18^ Promoters for each gene belonged to different transcription phases, and this promoter set resulted in higher GS productivity than the native state and all of the prepared plasmids for the library (Figure 1). These results demonstrate that the optimization of promoters can fine-tune the lethal molecule production phase to the best phenotypic resistance phase to create producers over the lethal level. This fine-tuning procedure can be effectively conducted using the Combi-OGAB screening cycles. In addition, to examine which promoter is important for producing or suppressing byproducts, each promoter of 3rd-C2 was replaced with *P*_*srfAA*_ (*P*_*srfAA*_-GS: byproduct producer). Byproducts were obtained only when the promoter for *grsB* (*P*_*lytR*_) was replaced with *P*_*srfAA*_ (Figure S3), indicating that the substrate selectivity for ^L^Orn against ^L^Lys was dominated only by the promoter for *grsB*.

In addition, we also monitored the time profiles of growth and GS productivity of the screened 3rd-C2 and selected material clones for the library construction (Figure S4). We selected *P*_*valS*_-GS, *P*_*srfAA*_-GS, *P*_*sigW*_-GS, and 3rd-C2 for this monitoring. 3rd-C2 was selected as the highest producer in this study, and the three clones (*P*_*valS*_-GS, *P*_*srfAA*_-GS, and *P*_*sigW*_-GS) were selected material clones with varied GS productivity including the non-producer (*P*_*valS*_-GS) and the best producer (*P*_*sigW*_-GS) among the materials, described in Figure 2. By 28 h, the level of GS production by 3rd-C2 reached a maximum, whereas *P*_*srfAA*_-GS and *P*_*sigW*_-GS showed subsequent production after 28 h. Moreover, none of the clones exhibited GS productivity higher than that of 3rd-C2. Notably, the initial production rate of 3rd-C2 was higher than that of *P*_*sigW*_-GS. In terms of promoters for *grsA* and *grsB*, those of 3rd-C2 belong to earlier transcription class than those of *P*_*sigW*_-GS.^18^ Thus, GrsA and GrsB (core enzymes for GS biosynthesis) are considered to be likely synthesized in 3rd-C2 earlier than in *P*_*sigW*_-GS, accounting for the relatively accelerated initial GS production rate of 3rd-C2 compared to that of *P*_*sigW*_-GS. These results suggest that the promoter combination in 3rd-C2 was precisely optimized toward higher GS productivity via Combi-OGAB.

## Discussion

In this study, we successfully created heterologous GS producers with the top producer synthesizing GS at levels approximately 30-fold higher than the lethal concentration of exogenous GS. *A. migulanus* accumulates GS inside the cell, and the produced GS can be extracted from a disrupted cell solution.^24^ We collected GS from a culture of heterologous GS producers after biosynthesis using ethyl acetate, indicating that our producers secrete GS outside the cell. To the best of our knowledge, there have been no reports on GS secretory producers; this is the first report of the production of GS secretory producers with the secreted concentration exceeding the lethal concentration. Moreover, the culture of 3rd-C2 after GS production was centrifuged and filtered, and the solution was mixed with LB medium at various ratios. The host *B. subtilis* and 3rd-C2 were then cultured in these media, although these media contained lethal concentrations of GS (Figure S5). A previous report demonstrated that exogenous GS is detoxified by surfactin through molecular interactions between cationic GS and anionic surfactin.^10^ We also observed that 3rd-C2 can grow in exogenous GS-containing medium detoxified by exogenous surfactin (Figure S6). Surfactin is natively produced by *B. subtilis* (Figure S3); therefore, this detoxification mechanism may have one of the roles to confer GS productivity with a lethal level on 3rd-C2.

In addition, overexpression of *botT*, which encodes a multidrug transporter, significantly enhanced the productivity of the antibacterial peptide bottromycin.^25^ Many BGCs often contain genes encoding efflux transporters, which are involved in the detoxification against the produced antibiotics.^26^ For the heterologous production of antibiotics with no existing exporters such as GS, our method holds the potential to create heterologous producers for these lethal antibiotics.

The screening cycles demonstrated here offer the possibility of achieving heterologous production from BGCs identified through metagenomic sequences, enabling the acquisition of their lethal molecules, despite their toxicity toward the production hosts. In the present study, we selected GS as a model antibiotic and *B. subtilis* as a model heterologous host to demonstrate our method. Although surfactin production by *B. subtilis* may have a role in the resistance against GS, other mechanisms could contribute to GS production by *B. subtilis*. Without any information about resistance in advance, our method holds possibility to create heterologous producers for lethal antibiotics. Therefore, we expect that this approach will be a starting tool for detecting and identifying the products of mined BGCs and contribute to the discovery of novel antibiotics.

## Materials and Methods

### Bacterial Strains, Plasmids, and Medium

The *E. coli* JM109 strain was used for the construction of OGAB blocks, and the *B. subtilis* BUSY9797 strain was used for the assembly of OGAB blocks into plasmid DNA. The pBR322 Δ*TypeIIS* IIS and pGETS151 Δ*BsmBI* plasmids were used for *E. coli* and *B. subtilis*, respectively. The pUB8 plasmid containing the *lpa-8* gene was used to express phosphopantetheinyl transferase.^13^*E. coli* was cultured in an LB medium containing 100 μg/mL carbenicillin, and *B. subtilis* was cultured in an LB medium containing 10 μg/mL tetracycline for *grsT*-*grsA*-*grsB* construction. GS production by *B. subtilis* was conducted in the YTG medium containing yeast extract (50 g/L), Bacto tryptone (50 g/L), glucose (5 g/L), tetracycline (10 μg/mL), and kanamycin (10 μg/mL). The transformation procedures for *B. subtilis* have been previously reported.^14,15^

### Design of a GS Biosynthetic Gene Cluster

The GS BGC of *A. migulanus* DSM2895 (non-GS producer^24,27^) was used for sequence design. Native *grsA* and *grsB* in *A. migulanus* have recognition sequences for restriction enzymes AarI, BsaI, BbsI, BsmBI, and BspQI, and silent mutations have been introduced into them. The 20-, 22-, or 20-bp sequences upstream of native *grsT*, *grsA*, and *grsB*, respectively, were attached upstream of each gene. These sequences included the Shine-Dalgarno (SD) sequence from *A. migulanus*.^6^ The SfiI recognition sequences and promoter introduction sites were also attached upstream of each SD sequence. Promoter introduction sites were used for the ligation of the terminator–promoter cassettes after the construction of the designed BGC. SfiI recognition sequences were used to construct a combinatorial promoter library using Combi-OGAB. The entire designed GS BGC was 17,669 bp long.

### Synthesis of GS Biosynthetic Gene Clusters with Phase-Dependent Promoters

The designed sequence was divided into 25 OGAB blocks, which were assembled on the pGETS151 Δ*BsmBIB. subtilis* vector using the OGAB protocol.^15^ The constructed sequences (Supporting Information) were determined using MiSeq (Illumina). Phase-dependent promoters have been previously described.^18^ The ρ-factor-independent terminator BBaK_780000 is listed in the iGEM. Promoters were cloned from the Marburg168 genome, and they were combined to the 3′-end of BBaK_780000. Each BBaK_780000-promoter cassette (Table S2) was constructed using the pBR322 Δ*TypeIIS* plasmid. These cassettes were amplified by PCR, and the PCR products were digested with PaqCI. To introduce these cassettes upstream of *grsT*, *grsA*, or *grsB*, the constructed plasmids were digested with PaqCI, BsaI, or BbsI, respectively. The digested linear DNA was ligated to the promoter cassettes. These plasmids were used to construct an initial library. To introduce *P*_*grs*_ into *grsT*, the plasmid was digested with PaqCI, and *P*_*grs*_ (Table S2) was ligated using the same procedure. This construct was used in its native state (*P*_*grs*_-GS; Figure 1).

### Construction of a Combinatorial Promoter Library

The 10 prepared plasmids were mixed equally and digested with SfiI at 50 °C. After complete digestion, the enzyme was deactivated with phenol, and the solution was concentrated with 1-butanol. DNA was then precipitated with ethanol and re-dissolved in a TE buffer. SfiI-digested DNA was ligated with T4 DNA Ligase at room temperature, and the ligated product was used for the transformation of BUSY9797 with pUB8. Transformants were plated on an LB plate containing 10 μg/mL tetracycline and 10 μg/mL kanamycin, and the plate was incubated at 30 °C overnight. Single colonies were individually picked and cultured in 300 μL/well of the LB medium containing 10 μg/mL tetracycline and 10 μg/mL kanamycin in a 96-well deep-well culture plate. The plates were shaken at 30 °C overnight. These plates were stored at −70 °C until GS productivity analysis.

### Analysis of GS Productivity

A small aliquot of frozen cells was directly inoculated into 2 mL of the YTG medium and shaken at 30 °C for 72 h. Each 72 h culture was mixed with 2 mL of ethyl acetate for 30 s on a vortex mixer. The ethyl acetate fraction was evaporated to dryness, and the residue was redissolved in 200 μL of 70% methanol containing 0.05% formic acid. Analytical HPLC (COSMOSIL 5C18-AR-II packed column 4.6 mm I.D. × 150 mm, 1.0 mL/min, 210 nm) was performed in a 0.1% formic acid–water (solvent A) and methanol (solvent B) gradient. The gradient was run from 60% solvent B for 0–5 min, 60 to 78% solvent B for 5–14 min, 78 to 100% solvent B for 14.0–14.1 min, and 100% solvent B for 5 min. GS was detected at *t*_*R*_ = 12.8 min using a gradient program. An authentic GS sample was purchased from AbMole and used to quantify GS productivity based on the area value (mAU × min) on HPLC. The same GS solution was used for the exogenous GS sensitivity assay as described below.

### Preparation of the 2–4th Combi-OGAB Screening Cycles

Selected producers were re-inoculated in 2 mL of the LB medium containing 10 μg/mL tetracycline and 10 μg/mL kanamycin from the frozen stock in each cycle. Equal volumes of the cultures were mixed, and the plasmid DNA was extracted. The plasmid mixture was digested with SfiI, and the digested DNA was ligated for the next cycle. The following procedures were performed, as described above.

### LC-MS Analysis of GS and Byproducts Produced by the Screened Producer

The extracted sample of 3rd-C2 clone was analyzed by using LC-MS. Analysis (machine: ACQUITY UPLC Class H, MS: Xevo G2-QTOF, column: ACQUITY UPLC BEH C18 [50 × 2.1 mm I.D., 1.7 μL], injection: 1 μL, Solvent: 0.1% formic acid–water [A] and 0.1% formic acid-acetonitrile [B], gradient: 30 to 100% B in 0 to 10 min and 100% B in 10 to 12 min, flow rate: 0.8 mL/min, column temperature: 55 °C, wavelength: 210–500 nm, ionization method: ESI, orifice voltage: 25 eV) was performed by OP Bio Factory Co., Ltd. (Okinawa, Japan).

### Determination of Promoter Sequences in Selected Clones

The three promoter positions in 3rd-C2 construct were amplified using suitable promoter sets (Table S3). The promoter sequences of each fragment were analyzed using a 3150xL DNA sequence analyzer (Applied Biosystems).

### Analysis of the Viability of the Producer against Exogenous GS

BUSY9797 and 3rd-C2 were pre-cultured in 2 mL of LB without antibiotics or with 10 μg/mL tetracycline and 10 μg/mL kanamycin, respectively, at 30 °C for overnight. A small aliquot was subcultured in 2 mL of LB containing 0 (acetonitrile), 1, 5, 10, or 30 μg/mL GS diluted from GS-acetonitrile stock solution. They were cultured at 30 °C for 24 h, and their growth was monitored.

## Supporting Information Available

Figure S1: LC-MS spectra of the produced fraction including GS and byproducts, Figure S2: Viability of the host *B. subtilis* and 3rd-C2 in the LB medium containing GS, Figure S3: HPLC chromatograms of the extracted samples of 3rd-C2 and *P*_*srfAA*_-substituted 3rd-C2, Figure S4: Time profiles of growth and GS production of 3rd-C2, *P*_*valS*_-GS, *P*_*srfAA*_-GS and *P*_*sigW*_-GS, Figure S5: Viability of the host *B. subtilis* and 3rd-C2 in the LB medium containing a filtrated culture of 3rd-C2 after GS production, Figure S6: Viability of the host *B. subtilis* and 3rd-C2 in the LB medium containing 1 μg/mL GS and 63.7 μg/mL surfactin, Table S1: Number of clones analyzed in Combi-OGAB screening cycles, Table S2: Sequences of terminator-promoter cassettes, and Table S3: Primer sequences for DNA sequencing and sequence of the constructed pGETS151 Δ*BsmB*I-GS BGC (PDF)
