# Supplementary material for: A Novel Method for Creating Heterologous Lethal Antibiotic Producers by Screening from Combi-OGAB Library with Various Promoters in a Biosynthetic Gene Cluster
Source: ACS Omega. 2024 Jan 30;9(6):6873–9. doi: 10.1021/acsomega.3c08240 (PMC10870263; doi:10.1021/acsomega.3c08240)
Supplement: Supplementary file 1 — ao3c08240_si_001.pdf [file ao3c08240_si_001.pdf]

Supporting information for

A novel method for creating heterologous lethal antibiotic producers by screening from Combi-OGAB library with various promoters in a biosynthetic gene cluster.

Naoki Miyamoto*, Akinori Nishigami, Nao Hosoda, Kentaro Hayashi, Naoyuki Yamada, and Kenji Tsuge

Synplogen Co., Ltd., Hyogo, Japan

E-mail: n_miyamoto@synplogen.com, orcid.org/0000-0001-5544-191X

Supplemental Figures

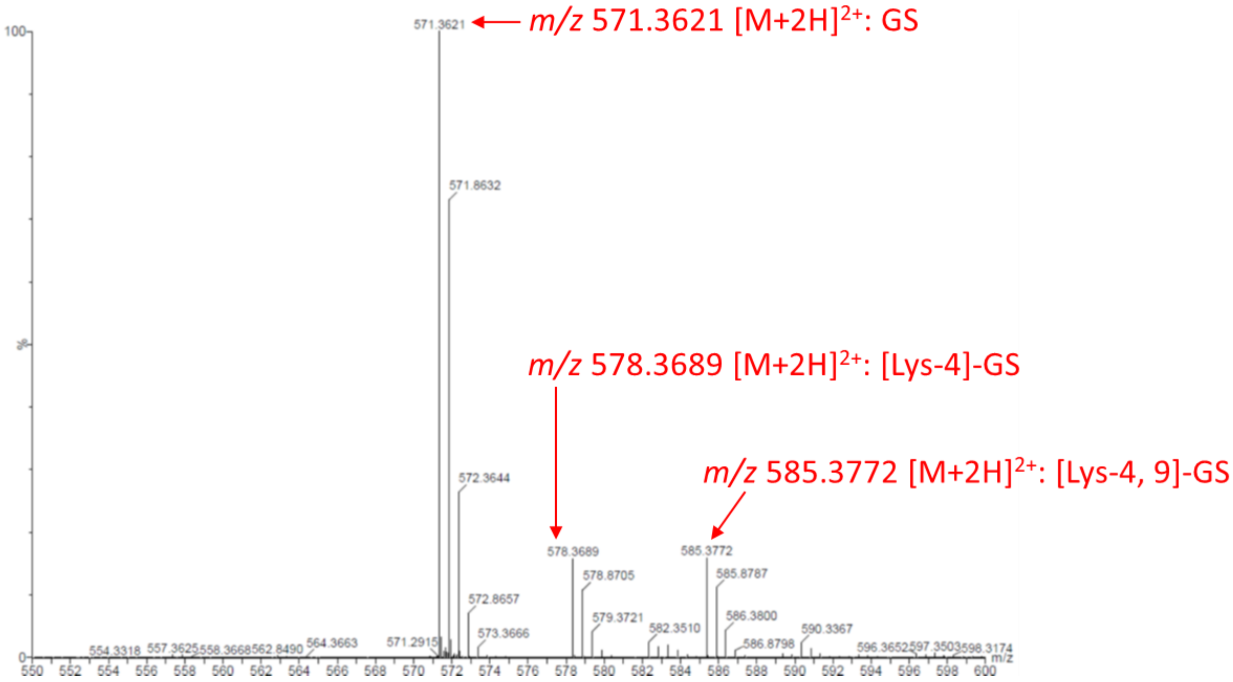

Figure S1. LC-MS spectra of the produced fraction including GS and byproducts (t_R=3.39 min). Three peaks of *m/z* were detected, and *m/z* 571, 578, and 585 ([M+2H]$^{2+}$) of the peaks correspond to GS, [Lys-4]-GS, and [Lys-4, 9]-GS, respectively.

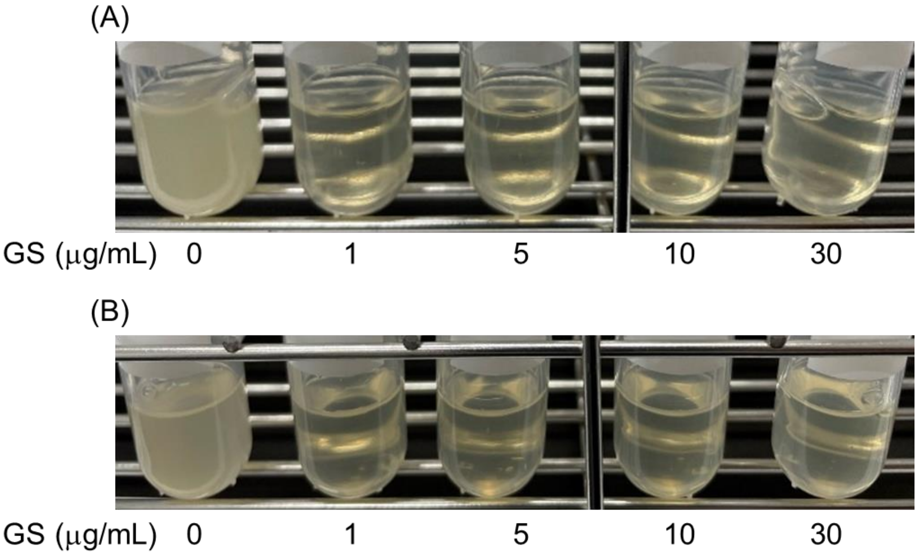

Figure S2. Viability of (A) the host bacterium *B. subtilis* BUSY9797 and (B) 3rd-C2 in LB solution medium containing exogenous GS. A small aliquot of overnight culture was sub-cultured into 2 mL LB containing GS = 0 (acetonitrile), 1, 5, 10, or 30 μg/mL, diluted from GS-acetonitrile stock solution. They were cultured at 30 °C for 24 h.

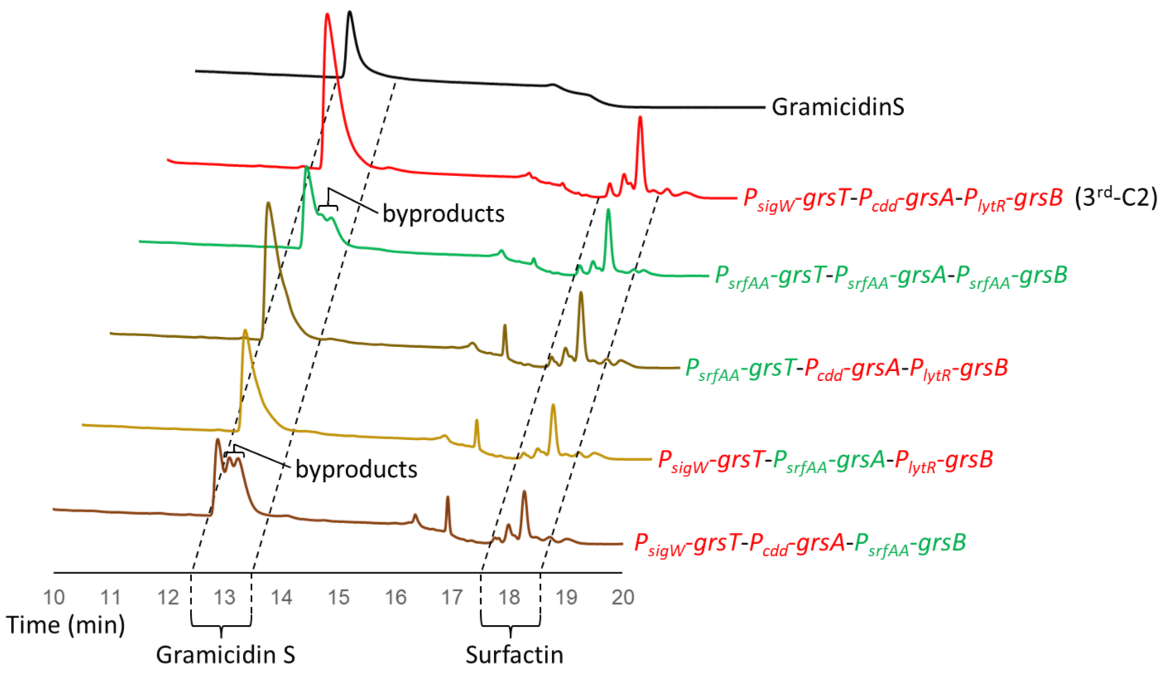

Figure S3. High-performance liquid chromatogram of GS (2 μg) and extracted samples of 3rd-C2 and P_{srfAA} -substituted 3rd-C2. Only when the promoter for *grsB* of 3rd-C2 (P_{lytR}) was exchanged to P_{srfAA}, was byproduct productivity substantially increased (the bottom chromatogram). The productivity of GS and byproducts in 3rd-C2, P_{srfAA}-*grsT*-P_{srfAA}-*grsA*-P_{srfAA}-*grsB*, P_{srfAA}-*grsT*-P_{cdd}-*grsA*-P_{lytR}-*grsB*, P_{sigW}-*grsT*-P_{srfAA}-*grsA*-P_{lytR}-*grsB* and P_{sigW}-*grsT*-P_{cdd}-*grsA*-P_{srfAA}-*grsB* is 29.7 mg/L, 19.0 mg/L, 26.7 mg/L, 18.8 mg/L and 20.2 mg/L, respectively.

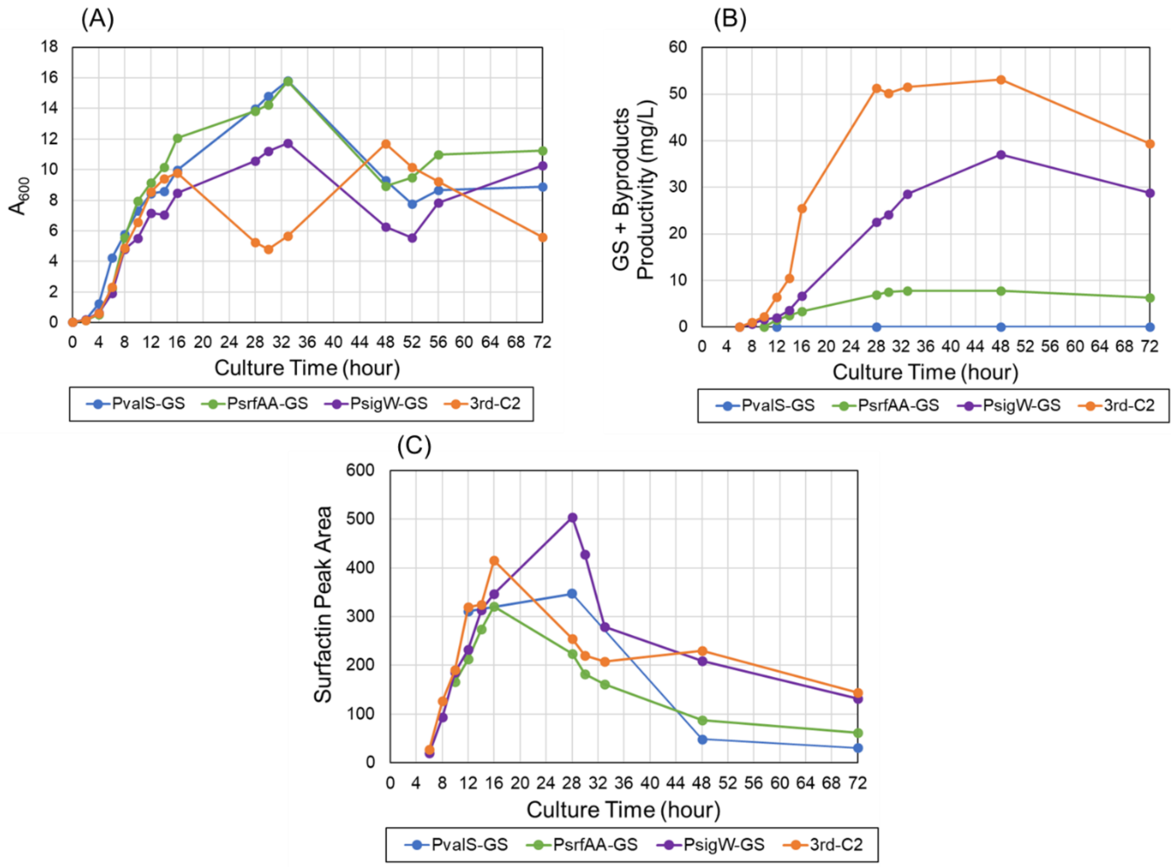

Figure S4. Comparison of four *B. subtilis* clones containing P_{valS}-*grsT*-P_{valS}-*grsA*-P_{valS}-*grsB* (P_{valS}-GS), P_{srfAA}-*grsT*-P_{srfAA}-*grsA*-P_{srfAA}-*grsB* (P_{srfAA}-GS), P_{sigW}-*grsT*-P_{sigW}-*grsA*-P_{sigW}-*grsB* (P_{sigW}-GS), or P_{sigW}-*grsT*-P_{cdd}-*grsA*-P_{lytR}-*grsB* (3rd-C2). (A) Time profiles of cell growth. Each clone was inoculated into 2 mL of LB medium from the frozen stock and cultured at 30 °C overnight. A small aliquot with the same bacterial density was sub-cultured into 2 mL of YTG medium and cultured at 30 °C overnight. A small aliquot of the same bacterial density was sub-cultured into 100 mL of YTG medium and cultured at 30 °C for 72 h. A small aliquot of each culture was collected at different time points during the culture period, and bacterial density (A_{600}) was analyzed. (B) Time profiles of total GS (GS and byproducts) productivity. At the same time points for the bacterial density analysis, 2 mL of each culture was collected, and GS was extracted, and GS productivity was analyzed using high-performance liquid chromatography. (C) Detected surfactin amounts produced by each clone on HPLC. In the same extraction and analysis procedures, surfactin was also detected on HPLC. Compared with GS, production rate of surfactin is faster, and the production rate among four clones was not different. The peak area value of 400 is equivalent to approximately 338.7 mg/L of surfactin.

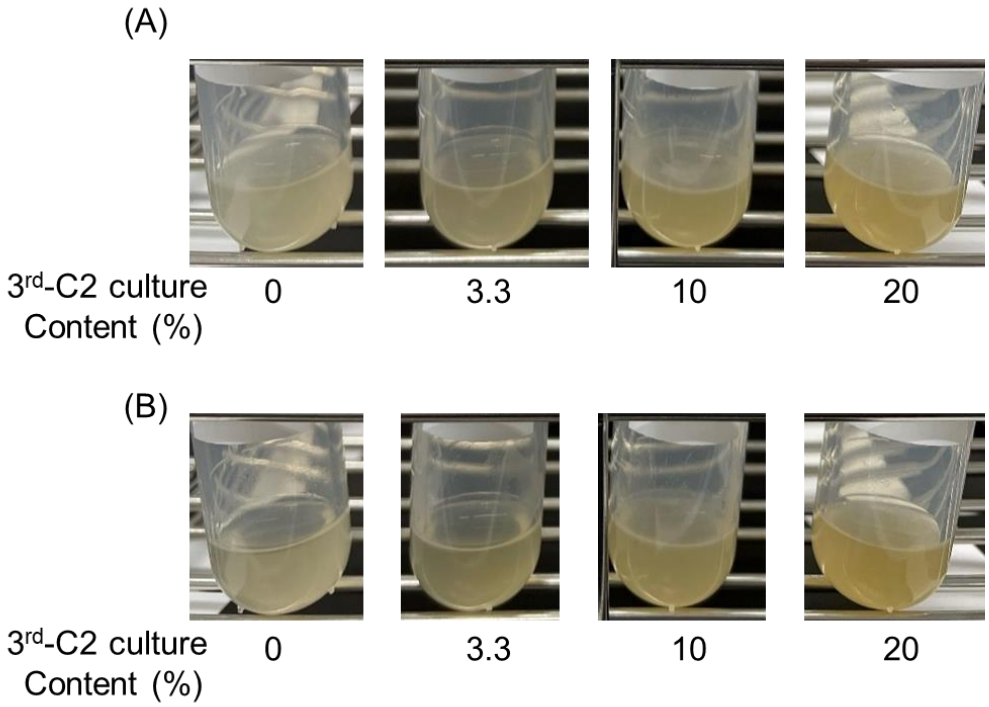

Figure S5. Viability of (A) the host bacterium *B. subtilis* BUSY9797 and (B) 3rd-C2 in LB medium containing a filtrated culture of 3rd-C2 after GS production. A small aliquot of overnight culture was sub-cultured into 2 mL LB containing 3.3, 10, or 20% of filtrated 3rd-C2 72-h culture. They were cultured at 30 °C for 24 h.

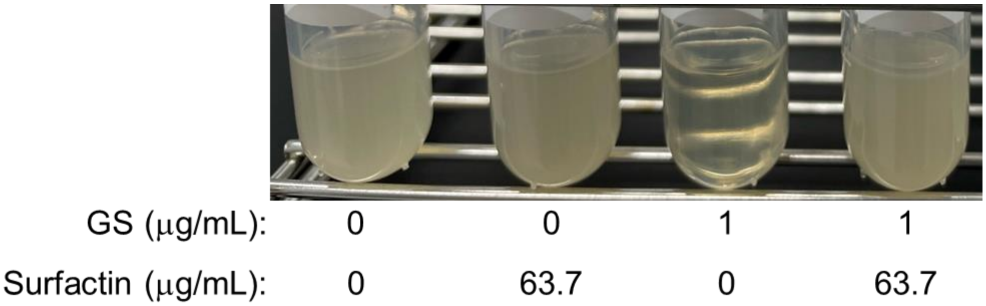

Figure S6 Viability of 3rd-C2 in LB medium containing 1 μg/mL GS and/or 63.7 μg/mL surfactin. A small aliquot of overnight culture was sub-cultured into the above medium and cultured for 72 h.

Supplemental Tables

Table S1. Number of clones analyzed in Combi-OGAB screening cycles.

	1st Cycle	2nd Cycle	3rd Cycle	4th Cycle
Selected clones for library construction	-	7	7	5
Transformants (cfu)	> 10,000	> 10,000	> 10,000	> 10,000
Picked clones	192	60	30	20
Culturable clones	134	59	30	20
GS producers	65	45	30	20
Byproduct producers	12	2	0	0

Table S2. Terminator-promoter cassettes used in this study.

Name	Sequence (5′ to 3′)
BBaK_780000-P_{valS}	ATCGaagaagaagtgtgaaaaagcgcagctgaaatagctgcgctttttgtgtcataaTCCTAG<br>ATTAGGATTGCGAGCAGCAAAACATCAAAGGTTGTCGGAAAG<br>AAAATGGTTCTGATTCCTTGAAAAATCGTAATGGGAATAATCA<br>CTTGTGCGGATACAAGCCGAATTTGCCTTACCCATGGCGGCAG<br>CGAATAAGAGCTGAAACGTTTCTTCACGGTGCATCCTCCTCTA<br>CGCATTTACAAGCATCATATGTAAACTTGGGCAATAGGTGCCT<br>GCCCATCTGTATAGAAAGCGGTGTTTTTGGAAAAGATGATTGA<br>CGAATCAAAAACACTTTTACTATAATAAAGAACATAAATAAGG<br>AA
BBaK_780000-P_{hag}	ATCGaagaagaagtgtgaaaaagcgcagctgaaatagctgcgctttttgtgtcataaTCCTTG<br>AAGTAAAAGTGATTGCGGTTGAAGGGGATCAAGTGAAGCTTG<br>GAATTGACGCCCCAAAGCATATTGATATTCACAGGAAAGAAAT<br>TTACTTGACCATTCAGGAAGAAAATAACCGTGCAGCAGCGTTA<br>TCCAGCGATGTGATCTCCGCATTATCCTCACAAAAAAAGTGAG<br>GATTTTTTTATTTTTGTATTAACAAAATCAGAGACAATCCGATA<br>TTAATGATGTAGCCGGGAGGAGGCGCAAAAGACTCAGCCAGT<br>TACAAAATAAGGGCACAAGGACGTGCCTTAACAACATATTCG<br>GAA
BBaK_780000-P_{spoVG}	ATCGaagaagaagtgtgaaaaagcgcagctgaaatagctgcgctttttgtgtcataaTCCTTG<br>CGGAAGTAAACGAAGTGTACGGACAATATTTTGACACTCACAA<br>ACCGGCGAGATCTTGTGTTGAAGTCGCGAGACTCCCGAAGGAT<br>GCGTTAGTCGAGATCGAAGTTATTGCACTGGTGAAATAATAAG<br>AAAAGTGATTCTGGGAGAGCCGGGATCACTTTTTTATTTACCTT<br>ATGCCCGAAATGAAAGCTTTATGACCTAATTGTGTAACTATAT<br>CCTATTTTTTCAAAAAATATTTTAAAAACGAGCAGGATTTCAG<br>AAAAAATCGTGGAATTGATACACTAATGCTTTTATATAGGGAA
BBaK_780000-P_{srfAA}	ATCGaagaagaagtgtgaaaaagcgcagctgaaatagctgcgctttttgtgtcataaTCCTGA<br>CGCTCTTCGCAAGGGTGTCTTTTTTTGCCTTTTTTTCGGTTTTTG<br>CGCGGTACACATAGTCATGTAAAGATTGTAAATTGCATTCAGC<br>AATAAAAAAAGATTGAACGCAGCAGTTTGGTTTAAAAATTTTT<br>ATTTTTCTGTAAATAATGTTTAGTGGAAATGATTGCGGCATCCC<br>GCAAAAAATATTGCTGTAAATAAACTGGAATCTTTCGGCATCC<br>CGCATGAAACTTTTCACCCATTTTTCGGTGATAAAAACATTTTT<br>TTCATTTAAACTGAACGGTAGAAAGATAAAAAATATGGAA
BBaK_780000-P_{cdd}	ATCGaagaagaagtgtgaaaaagcgcagctgaaatagctgcgctttttgtgtcataaTCCTTA<br>TTGAGTGGATGATTATATTCCTTTTGATAGGTGGTATGTTTTCG<br>CTTGAACTTTTAAATACAGCCATTGAACATACGGTTGATTTAAT<br>AACTGACAAACATCACCCTCTTGCTAAAGCGGCCAAGGACGCT<br>GCCGCCGGGGCTGTTTGCGTTTTTGCCGTGATTTCGTGTATCAT<br>TGGTTTACTTATTTTTTTGCCAAAGCTGTAATGGCTGAAAATTC<br>TTACATTTATTTTACATTTTTAGAAATGGGCGTGAAAAAAAGC<br>GCGCGATTATGTAAAATATAAAGTGATAGCGGTACCGGAA

BBaK_780000-P_{lytR}	ATCGaagaagaagtgtgaaaaagcgcagctgaaatagctgcgcttttttgtgtcataaTCCTCT<br>AACCCTACATAAGTACCTTCTTTTGTTTCAATGTTACTGTCTGG<br>CGATACATCTTCACCTTGACTCTTTTGACTATTAACCCCGCAAC<br>CCGAAAGAAGCAATATAAAGAACAGTAAAGCAATAAATTTTT<br>TCATTTTTTTCACCTCATTATATTTTATCGTCAACCTATTTTATA<br>TTTTAAAGAAAAATTAAGAAACAATGAAACTTTTTTTTATAAA<br>AAACGACTATTTTAGGATTTCATTCTTGTATTAAATAGAGTTGT<br>ATTTATTGGAAATTTAACTCATAATGAAAGTAATTTGGAA
BBaK_780000-P_{veg}	ATCGaagaagaagtgtgaaaaagcgcagctgaaatagctgcgcttttttgtgtcataaTCCTTA<br>TGGGAAGTGCTCCGTAATACGCTGACAAGAGAGAAAGGGCTT<br>GGAGGTATTGAAACAAGAGGAGTTCTGAGAATTGGTATGCCTT<br>ATAAGTCCAATTAACAGTTGAAAACCTGCATAGGAGAGCTATG<br>CGGGTTTTTTATTTTACATAATGATACATAATTTACCGAAACTT<br>GCGGAACATAATTGAGGAATCATAGAATTTTGTCAAAATAATT<br>TTATTGACAACGTCTTATTAACGTTGATATAATTTAAATTTTAT<br>TTGACAAAAATGGGCTCGTGTTGTACAATAAATGTAGTGGGAA
BBaK_780000-P_{mmgA}	ATCGaagaagaagtgtgaaaaagcgcagctgaaatagctgcgcttttttgtgtcataaTCCTTC<br>GATATATCGCGTCTATTCCGGCTTCCGGCTATCACCCGAAGAT<br>AAACAGCCCAGGGGTCACAGATGAAGTACTGAAGAAAATGAG<br>GAACGGTTTGATTAAGGTAAGGCCGTATACAGTCAATCGTCCG<br>GAAGATATGAAGCGTCTCATTGAAGCGGGAGCAGACGGCATG<br>TTTACCGACTTTCCAGAAAAGGCTTCGGCATTGCTGAAAAATG<br>AATAGTTGTTAGAAGGAGGCTGTTTGACGCAGCCTTCTTTTTTC<br>ATTCATTCATGCCCGTTTCAAAGCATACATTCATAGAAGACGG<br>AA
BBaK_780000-P_{yqfD}	ATCGaagaagaagtgtgaaaaagcgcagctgaaatagctgcgcttttttgtgtcataaTCCTAG<br>ATGCCCCTGCACCTATCCCTAACCGTATGGAACAGGCAAGACG<br>GGAAGCGGAAGAAAGACGCAGGGAAACAGCAAGAAACCTGA<br>AAGGGCTGGAACGAGATCTTGCTGCTGCCAAACAAAAAACAG<br>TATACACAAAACAAAAAATGCTTCAGGTGAATAAAGACACCG<br>TCGTACAGGGGATCGTTCTAGGAGAGGTGTTCGGACCTCCACG<br>GGCGAAAAAACCTCACCGTACGATGCGCCCGGCCCGTAAAAA<br>TTAAAGTGTTAGAACCTCCTTTCAAATCATACATATGAGATGA<br>AAGGAA
BBaK_780000-P_{sigW}	ATCGaagaagaagtgtgaaaaagcgcagctgaaatagctgcgcttttttgtgtcataaTCCTTG<br>CCCCCCTCCACCATTATTGGGCTATAGCCAAGCGGTAAGGCAA<br>CGGACTTTGACTCCGTCATGCGTTGGTTCGAATCCAGCTAGCC<br>CAGTCACAGACACCTTTGATCAAAAGGTGTCTTTTTTCTTTTCG<br>GAAAAATCATTCCAACTTCTAACTGTTCAGTCTGTATAATAATT<br>TTAAAAATATGTTAAGGTAGTTTATTCACGAATTACCATCTAC<br>ACCCTGCCAAAAATTTGATAAACTTATTTTATAAAAAAATTGA<br>AACCTTTTGAAACGAAGCTCGTATACATACAGACCGGTGGAA
Pgrs	ATCGTTACTGTATTGAAAAAAAATGTAAAATCGAATAAGCTTA<br>ACTCGAATCAGGTAAATAATGTATTAAAGTGATTTTTCTTCCAT<br>CCTGTGTAATTCAGCCAGCGAAATTTAAATTTGAAAGATAGTA<br>TTATTACTTTACTTATATATTATATAAGCAAGGAAAAATAAAG

	AATTGGCTGCCTCAAGATTTAAACATACTACATTTATCCATTCG GAATTCACTTCATAAGCAATTTATTCTTACATATATTTTTGCGT GATTTAATTTATTAATTAGATATTAAAAATGAATAAAGGGAGA GGTGAATGGAA

*Small letter: BBaK_780000 terminator

*5′-ATCG and 3′-GGAA: protruding ends for introduction into the plasmid

Table S3. List of primers used in this study.

Primer Name	Sequence (5′ to 3′)
grsT-promoter-seq-F	GGTCAACTACCCACCACTT
grsT-promoter-seq-R	CGCCTGCATATGGAATACAGA
grsA-promoter-seq-F	GTGTGTATGTATCCAGGTGATCA
grsA-promoter-seq-R	GGTGTTGTTCACAGCAAAGAGA
grsB-promoter-seq-F	GAAGGAAGATACTGAGTTAACTCCA
grsB-promoter-seq-R	GAGCATTTTTATCTTTATCAAGTAATGCGTGA

pGETS151 Δ*BsmB*I-GS BGC

AAAAGGCCTTCTTGGCCGCCCTTCCCGGTCGATATGAACAGCTTATTTACATAATTC
ACGTTATTGGTAGTTATAAATGAAATTCCTAATATCGGTTATGAAGTGAAATTGAAT
TTCTACTTGATCTTTCTCTCTATTTTTGTAAAATAAAATTAAGAATATTTAAATATTC
AATGATTCATTTTTGCAGAAATCGGAGGAAGAAGAATATATGAAAACATTTAACATT
TCTCAACAAGATCCCCCATATTGTTGTATAAGTGATGAAATACTGAATTTAAAACC
TAGTTTATATGTGGTAAAATGTTTTAATCAAGTTTAGGAGGAATTAATTATGAAGTG
TAATGAATAATGAGTGTAACAGGGTTCAATTAAAAGAGGGAAGCGTATCATTAACC
CTATAAACTACGTCTGCCCTCATTATTGGAGGGTGAAATGTGAATACATCCTATTCA
CAATCGAATTTACGACACAACCAAATTTTAATTTGGCTTTGCATTTTATCTTTTTTTA
GCGTATTAAATGAAATGGTTTTGAACGTGTCATTACCTGATATTGCAAATGATTTTA
ATAAACCACCAGCGAGTACAAACTGGGTGAACACAGCCTTTATGTTAACCTTTTCCA
TTGGAACAGCTGTATATGGAAAGCTATCTGATCAATTAGGCATCAAAAGGTTACTCC
TATTTGGAATTATAATAAATTGTTTCGGGTCGGTAATTGGGTTTGTTGGCCATTCTTT
CTTTTCCTTACTTATTATGGCTCGTTTTATTCAAGGGGCTGGTGCAGCTGCATTTCCA
GCACTCGTAATGGTTGTAGTTGCGCGCTATATTCCAAAGGAAAATAGGGGTAAAGC
ATTTGGTCTTATTGGATCGATAGTAGCCATGGGAGAAGGAGTCGGTCCAGCGATTGG
TGGAATGATAGCCCATTATATTCATTGGTCCTATCTTCTACTCATTCCTATGATAACA
ATTATCACTGTTCCGTTTCTTATGAAATTATTAAAGAAAGAAGTAAGGATAAAAGGT
CATTTTGATATCAAAGGAATTATACTAATGTCTGTAGGCATTGTATTTTTTATGTTGT
TTACAACATCATATAGCATTTCTTTTCTTATCGTTAGCGTGCTGTCATTCCTGATATTT
GTAAAACATATCAGGAAAGTAACAGATCCTTTTGTTGATCCCGGATTAGGGAAAAA
TATACCTTTTATGATTGGAGTTCTTTGTGGGGGAATTATATTTGGAACAGTAGCAGG
GTTTGTCTCTATGGTTCCTTATATGATGAAAGATGTTCACCAGCTAGGTACTGCCGA
AATCGGAAGTGTAATTATTTTCCCTGGAACAATGAGTGTCATTATTTTCGGCTACATT
GGTGGGATACTTGTTGATAGAAGAGGTCCTTTATACGTGTTAAACATCGGAGTTACA
TTTCTTTCTGTTAGCTTTTTAACTGCTTCCTTTCTTTTAGAAACAACATCATGGTTCAT
GACAATTATAATCGTATTTGTTTTAGGTGGGCTTTCGTTCACCAAAACAGTTATATCA
ACAATTGTTTCAAGTAGCTTGAAACAGCAGGAAGCTGGTGCTGGAATGAGTTTGCTT
AACTTTACCAGCTTTTTATCAGAGGGAACAGGTATTGCAATTGTAGGTGGTTTATTA
TCCATACCCTTACTTGATCAAAGGTTGTTACCTATGGAAGTTGATCAGTCAACTTATC
TGTATAGTAATTTGTTATTACTTTTTTCAGGAATCATTGTCATTAGTTGGCTGGTTAC
CTTGAATGTATATAAACATTCTCAAAGGGATTTCTAAATCGTTAAGGGATCAACTTT
GGGAGAGAGTTCAAAATTGATCCTTTTTTTATAACAGGAATTGGGCATCAAATAAAA
CGAAAGACTGGGCCTTTCGTTTTATCTGTTGTTTGTCGGTGAACGCTCTCCTGAGTAG
GACAAGTCCGCCGGGAGCGGATTTGAACGTTGCGAAGCAACGGCCCGGAGGGTGGC
GGGCAGGACGCCCGCCATAAACTGCCAGGCATCAAATTAAGCAGAAGGCCATCCTG
ACGGATGGCCTTTTTGCGTTTCTACAAACTCTTCCTGTCGTCATATCTACAATTCTAC
ACAGCCCAGTCCAGACTATTGAATTGTATCACGGTTTTGATATCCTACCAATAACAA
ATTGATTGGAGGAATGCAAAGTGAATAATGAACCAGTAAAACGTGGTAAGAAGAAC
AGATGGGAATTAAACCTACCTATAATGACTTATGTAGTAGCTGATGATTGGATTGAT
AAACTAGGACACGAAACGTTTACTTTATGGTTGAGGTTCCATACTTGGGTAGATAGA
GAAGATGAACTCCGAGATTATGATCGCATACCTAGAAGTTTTGAGAACATATATAA
AAAGACACTAGGAATCTCAAAAAGTAAGTTTTATAGATTGATAAAACCTTTATGGG
AATATGGATTAATAGACATCATAGAATACGAAGAATCTAACCGTAATTCTACTAAAC
CTAAAAATATAATTGTTTATGAGTATCCTTTACACGAAATAGAAAGAAAGTATAAAC

CACTAGAAAAATTAAGAGATTGGGATAAAGACTATAATTCCGTTTCTAAAGAATTA
GGTAAAACAGGTGGTAGACCAAGGAAAAAAGATAGTGAAGAAGAACCCGAAAAGA
AACCCGAAGAAGTAACTAAAAAGAAACGTAAATATAAGTTAAAAAGAGTTATCCAC
AACGGTTTCAAAAATGAAACGGTGGAGGGTTTCAAAAATGAAACGGTGGAGGGTTT
CAAAAATGAAACGGTGACCGTTTCAAAAATAAAACCCAATAATTATTCAAATATCTT
TAATAACTTATCAAATATTTCTACTAATGTTTCAAATAATTTATTAATTGATGATGAT
GAGGAAATCGAAAATGAACCAACTGGTCGTACAATAAATAGGTCATTACTTTTTTCG
CAAGAAGATATTAAACAGGCCTATCAATTTATTAATAGATTTTCAGTTATACAGTTA
CGTGAAAACTTTAGCTTTGATAAACACTTTGAAGAACGGTTGGTATGTTATTTATGG
AAAGCAGGGATTTCTACTTTTTACACGCACGAAATCAGTAAAATGATAAAAAAAAT
AGCAGACTATGAAAAATCTAAAAAAGGTAGATTAAACCCAATACGTGACCGAGCCT
TATATATGGTAAATGGTCTTGTAATGAATAGAGCTTCTTCCCAAAGTGAACATGCTA
CTTATAAACTAAACCAATATAAAAAACAGAAGGAACAGGAAAAACAACAACAGGA
GCAACAAAGATCAAGAGTACCGTTCTATAATTGGTTGGAGGAAAGAGAAGAACAAA
CCGAAGGTCAACTACCCACCACTTAAGCGGCCGCAAGCTTGAAGAGCTCTTCTTTCA
GAACGCTCGGTTGCCGCCGGGCGTTTTTTATGAGATGTCTCGGCCTGTTTGGCCATTA
ATCGATCGGCAGGTGATGTCAATAACACCTGCATCGGGAAGAATAAAGGGAGAGGT
GAATGTGACTTTTATTTCACAAGTAAATAAATGGTTTGTTAATGCTAATGTTAACTCA
GCTGCAAAGCTTAGGCTATTCTGTATTCCATATGCAGGCGGTGGTGCTTCCGCCTTTT
ATGAATGGAGTCATTTTTTTCCAAAGGAAATTGAAGTTTGTTCAATTCAATTACCTG
GAAGGGAAAATAGGGGGGCGGAAGTTCCGCTAACAAATTTACAACAGATAGTAGA
AATAGTAGCTGAGGAAATACAACCATTAATAAATATTCCATTTGCTTTTTTGGGGCA
TAGCATGGGAGCATTAATAAGTTTTGAACTGGCTCGCACAATACGGCAAAAGAGTA
ATGTTAATCCGGTTCACTTGTTTGTTTCAGGGCGACATGCACCTCAAATCCCATGTGC
AAAACAAGACTATCATTTACTTCCCGATGAACAATTTATACAAGAATTGCGTTCATT
GAATGGAACTCCAGAGATAGTATTACAAGACGCAGAGATGATGAGTATATTACTCC
CAAGACTTCGGGCTGATTTTTCTGTGTGTGGCTCCTATCAGTACAAAAACGACGAGC
CTTTTGAATGCCCAATCACTGCTTTTGGAGGAAAAAATGATAATGGTGTTACTTATC
AATCATTAGAAGCCTGGAGAGAGCAAACCAAGAGGGAATTTTCTGTGTGTATGTAT
CCAGGTGATCATTTTTTTCTTTACGAAAGCAAATATGAAATGATTGAGTTCATGTGT
AAACAATTACGTTTAGTATTAGCTCCTAAAATATAAGGCCTTGATGGCCATCGAGAG
ACCAATAATAATAGGTCTCAGGAACTATTTTTTACAGGGGGTATATATGTTAAACAG
TTCTAAAAGTATATTGATTCATGCTCAAAATAAAAATGGAACGCATGAAGAGGAGC
AGTATCTCTTTGCTGTGAACAACACCAAAGCGGAGTATCCACGTGATAAGACGATCC
ATCAGTTATTTGAGGAGCAGGTTAGTAAGAGGCCAAACAATGTAGCCATTGTATGTG
AAAATGAGCAACTTACCTACCATGAGCTTAATGTGAAAGCCAATCAACTAGCACGG
ATTTTTATAGAAAAAGGGATTGGAAAAGACACTCTTGTTGGAATTATGATGGAGAA
ATCTATCGATTTATTTATAGGCATATTAGCCGTTTTAAAAGCTGGTGGAGCATATGTT
CCGATTGATATTGAATATCCTAAGGAAAGAATTCAATATATTCTTGATGATAGTCAG
GCAAGAATGCTACTTACCCAGAAGCATTTGGTTCATTTAATTCATAATATTCAATTTA
ATGGGCAAGTGGAAATTTTTGAAGAAGATACTATCAAAATTAGAGAAGGAACTAAT
CTACATGTACCAAGTAAATCAACCGATCTTGCTTATGTTATTTATACTTCTGGTACAA
CAGGCAATCCAAAAGGTACAATGCTGGAGCATAAAGGAATAAGTAATCTAAAGGTA
TTTTTCGAAAATAGTCTTAACGTGACTGAAAAGGATAGAATTGGTCAATTTGCCAGC
ATCTCTTTTGATGCATCTGTATGGGAGATGTTTATGGCTTTGTTAACGGGGGCTAGCC
TGTATATTATCCTGAAGGATACAATCAATGATTTTGTGAAGTTTGAACAATACATTA

ACCAAAAGGAAATCACTGTTATTACGTTACCACCTACCTATGTAGTTCATCTTGATC
CAGAACGTATTTTATCGATACAAACGTTAATTACAGCAGGCTCAGCTACCTCGCCTT
CCTTAGTAAACAAGTGGAAGGAGAAAGTAACTTACATAAATGCCTATGGCCCTACG
GAAACAACTATTTGTGCGACTACATGGGTAGCCACCAAAGAAACAATAGGTCATTC
AGTTCCAATCGGAGCACCAATTCAAAATACACAAATTTATATTGTCGATGAAAATCT
TCAATTAAAATCGGTTGGTGAAGCTGGTGAATTGTGTATTGGTGGAGAAGGGTTAGC
AAGGGGATATTGGAAGCGACCGGAATTAACTTCCCAGAAGTTCGTTGATAACCCGTT
TGTTCCAGGAGAGAAGTTGTATAAAACAGGAGATCAGGCAAGATGGCTATCTGATG
GAAATATTGAATATCTCGGAAGAATAGATAACCAGGTAAAGATTAGAGGTCACCGA
GTTGAACTAGAAGAAGTTGAGTCTATTCTTCTAAAGCATATGTATATTAGCGAAACT
GCAGTAAGTGTGCATAAAGATCACCAAGAACAGCCGTATTTGTGCGCTTATTTTGTA
TCGGAAAAGCATATACCACTAGAACAGTTAAGACAATTCTCATCAGAAGAACTGCC
AACGTATATGATCCCTTCTTATTTTATCCAGTTAGACAAAATGCCGCTTACATCAAAT
GGGAAGATTGATCGAAAGCAGTTGCCGGAACCTGATTTAACTTTCGGGATGAGGGT
AGACTATGAAGCGCCGCGAAATGAAATCGAGGAAACGCTTGTTACTATCTGGCAGG
ATGTATTAGGTATTGAGAAAATCGGTATTAAAGATAATTTCTATGCATTAGGTGGAG
ATTCTATTAAAGCAATACAGGTTGCTGCTCGCCTGCATTCCTACCAATTAAAGCTAG
AAACAAAAGATTTATTAAAGTATCCAACAATCGATCAACTCGTTCATTATATAAAAG
ATAGTAAAAGAAGAAGTGAGCAAGGTATTGTGGAAGGTGAGATTGGACTTACACCT
ATTCAGCATTGGTTCTTTGAACAACAATTTACAAATATGCACCATTGGAACCAATCG
TATATGTTGTATAGACCAAATGGGTTTGATAAAGAGATCTTGCTAAGGGTATTTAAT
AAAATTGTTGAGCATCATGATGCATTACGTATGATATACAAACATCATAACGGAAA
GATCGTGCAGATAAATCGGGGGCTTGAAGGTACGTTGTTTGATTTTTATACCTTTGA
TTTAACTGCAAATGATAATGAGCAACAGGTGATTTGTGAAGAATCTGCTCGATTACA
AAATAGTATAAACTTGGAAGTAGGCCCTCTAGTAAAGATAGCGCTGTTTCATACTCA
GAATGGAGATCACCTGTTTATGGCTATTCATCATTTGGTTGTGGATGGTATTTCTTGG
AGGATTTTGTTTGAGGATTTGGCCACAGCTTATGAACAAGCAATGCATCAGCAAACG
ATTGCTTTACCAGAGAAAACAGATTCATTTAAGGACTGGTCTATTGAATTAGAAAAA
TATGCGAACAGCGAATTATTCCTAGAAGAAGCTGAATATTGGCATCATTTGAATTAT
TATACCGAGAACGTTCAAATTAAGAAAGATTATGTCACCATGAACAATAAACAAAA
GAATATACGTTATGTAGGAATGGAGTTAACAATAGAAGAGACAGAAAAATTATTGA
AAAATGTAAATAAAGCGTATCGAACAGAAATTAATGATATTTTATTAACGGCACTTG
GCTTTGCACTCAAAGAATGGGCCGATATTGATAAAATTGTAATTAACTTAGAGGGAC
ACGGACGGGAAGAAATACTGGAACAGATGAACATTGCAAGGACGGTAGGCTGGTTT
ACTTCCCAGTATCCTGTTGTACTTGATATGCAAAAATCGGATGATTTGTCTTATCAAA
TCAAATTAATGAAAGAAAATTTACGCAGAATACCTAACAAAGGAATCGGATATGAA
ATTTTTAAGTATTTAACAACTGAATATTTACGGCCTGTTTTACCCTTTACATTAAAGC
CGGAAATTAACTTTAACTACTTAGGACAGTTCGATACGGACGTGAAAACTGAATTGT
TTACTCGTTCTCCTTATAGCATGGGTAATTCATTAGGACCAGATGGAAAAAATAATT
TAAGCCCAGAAGGGGAAAGTTATTTTGTACTCAATATTAATGGTTTTATTGAAGAAG
GTAAGCTTCACATCACCTTTTCTTATAATGAACAGCAGTATAAGGAGGATACCATTC
AGCAATTGAGCCGGAGCTATAAGCAACATCTTTTGGCCATCATTGAACATTGTGTAC
AGAAGGAAGATACTGAGTTAACTCCAAGTGATTTCAGTTTCAAGGAACTTGAATTAG
AAGAGATGGATGATATTTTCGATTTGTTGGCCGATTCATTAACGTAAGGCCTCGATG
GCCATCGATGTCTTCTTATTATTATGAAGACATGGAACATTATTATGAGGTGCTAGC
ATGAGTACATTTAAAAAAGAACATGTTCAGGATATGTATCGTTTATCTCCCATGCAG

GAAGGCATGTTGTTTCACGCATTACTTGATAAAGATAAAAATGCTCACCTGGTACAA
ATGTCTATCGCGATCGAAGGTATCGTGGATGTGGAGCTGCTTAGTGAAAGCTTGAAC
ATATTGATTGATAGATACGATGTGTTTAGAACAACATTCTTACATGAAAAAATTAAA
CAACCGCTTCAGGTAGTGCTAAAGGAACGGCCTGTTCAGCTTCAATTTAAAGACATA
TCATCCTTAGATGAAGAAAAAAGAGAACAGGCTATTGAGCAGTATAAGTATCAAGA
TGGGGAAACAGTCTTTGATTTAACAAGAGATCCCTTGATGAGAGTAGCTATTTTTCA
AACTGGTAAGGTTAACTACCAAATGATCTGGAGCTTCCACCATATTTTAATGGATGG
TTGGTGCTTCAACATTATATTTAATGACTTGTTCAATATATATCTGTCATTAAAAGAG
AAGAAACCTCTTCAGTTAGAGGCGGTGCAACCATATAAGCAGTTTATTAAGTGGCTT
GAAAAACAAGATAAACAGGAAGCACTTCGCTACTGGAAAGAACATTTAATGAATTA
TGATCAATCAGTAACATTACCTAAAAAGAAAGCAGCTATTAATAATACTACATATGA
ACCAGCACAGTTTCGTTTTGCGTTTGACAAAGTGCTTACCCAGCAGCTGCTTCGTATT
GCCAATCAAAGCCAAGTAACACTAAATATTGTTTTTCAAACAATATGGGGGATTGTA
CTTCAAAAATACAATTCCACTAATGATGTTGTATATGGCTCTGTTGTATCAGGCCGTC
CTTCTGAAATATCGGGAATTGAGAAAATGGTTGGACTATTTATTAATACTCTTCCATT
ACGTATCCAAACGCAAAAAGATCAATCATTTATTGAATTAGTAAAGACTGTTCATCA
AAACGTCCTTTTCTCGCAACAGCATGAGTATTTTCCATTGTATGAAATACAAAATCA
TACAGAATTAAAACAGAATCTGATTGATCATATTATGGTAATTGAAAATTATCCTTT
AGTAGAAGAATTGCAAAAGAATAGTATCATGCAAAAAGTAGGGTTTACAGTTCGTG
ATGTCAAAATGTTTGAACCAACTAATTATGATATGACAGTTATGGTTTTACCTCGTG
ATGAAATTAGTGTCCGACTCGATTATAACGCAGCCGTTTATGATATAGATTTCATAA
AAAAAATTGAAGGTCACATGAAAGAAGTGGCTTTATGCGTGGCAAATAATCCACAT
GTGTTAGTACAGGACGTTCCTCTGCTTACAAAGCAAGAAAAACAACATTTATTGGTA
GAGCTGCATGATTCGATAACAGAGTATCCTGATAAGACGATTCATCAGTTATTTACA
GAACAGGTAGAAAAAACACCAGAGCATGTGGCAGTTGTATTCGAAGATGAGAAAGT
GACCTATAGAGAGCTGCATGAGAGATCTAATCAATTAGCCAGATTCTTAAGAGAAA
AAGGCGTAAAAAAAGAAAGCATCATAGGCATTATGATGGAGCGTTCAGTTGAAATG
ATTGTTGGGATCTTAGGGATTTTAAAAGCTGGTGGAGCTTTTGTGCCTATTGATCCTG
AATATCCAAAAGAAAGAATCGGCTATATGTTAGATTCTGTACGGCTAGTACTTACAC
AACGCCATTTAAAGGATAAATTTGCTTTTACGAAAGAAACGATAGTAATTGAAGATC
CAAGTATTTCACACGAGTTAACTGAAGAAATAGATTATATTAATGAATCAGAGGACT
TGTTTTATATTATTTATACATCAGGAACAACAGGTAAACCAAAAGGGGTTATGCTAG
AGCACAAAAACATCGTTAATCTGCTTCATTTTACTTTCGAGAAAACAAATATCAACT
TTAGTGACAAAGTATTACAGTATACAACATGCAGTTTTGACGTGTGTTACCAAGAAA
TTTTTTCGACGCTCTTGTCTGGAGGGCAATTATATCTTATTAGGAAAGAAACTCAAC
GCGATGTAGAGCAATTATTTGATTTAGTAAAACGTGAAAATATTGAAGTATTATCCT
TTCCTGTGGCTTTTCTAAAATTTATTTTCAATGAAAGAGAATTTATCAATCGTTTTCC
AACTTGCGTGAAACATATTATCACAGCAGGAGAACAATTAGTAGTTAACAATGAGT
TTAAACGTTATTTGCATGAACATAACGTACATTTACACAATCATTATGGTCCATCAG
AAACGCATGTTGTTACCACCTATACTATTAATCCTGAAGCTGAAATTCCTGAATTAC
CACCGATAGGAAAACCTATCTCCAATACATGGATTTATATTTTGGATCAAGAACAAC
AACTACAACCACAAGGAATTGTAGGAGAGTTATATATTTCGGGCGCAAATGTTGGA
AGAGGATATTTGAATAATCAAGAATTAACGGCAGAAAAATTCTTTGCAGATCCCTTT
AGGCCAAACGAACGGATGTACCGAACAGGGGATTTAGCAAGGTGGTTGCCAGACGG
AAATATCGAATTTTTAGGAAGGGCCGATCATCAGGTGAAAATTAGGGGGCATCGAA
TAGAGCTTGGTGAGATCGAGGCACAATTATTAAATTGTAAGGGTGTAAAAGAAGCT

GTTGTTATCGATAAAGCGGATGATAAAGGCGGAAAATATTTATGTGCCTATGTTGTT
ATGGAAGTAGAAGTAAATGACTCTGAGCTTCGAGAATATTTGGGGAAAGCTTTGCCT
GATTATATGATCCCGTCGTTCTTTGTTCCGTTGGATCAGCTGCCGCTTACACCAAACG
GAAAAATAGACAGAAAATCTCTTCCGAATCTAGAGGGGATTGTGAATACAAACGCA
AAATATGTAGTACCTACAAATGAGCTGGAAGAAAAATTGGCTAAAATCTGGGAAGA
AGTACTTGGGATTTCTCAGATCGGTATACAAGACAATTTCTTTTCGTTAGGCGGGCA
TTCTCTTAAAGCCATTACGCTTATTTCCCGTATGAACAAAGAGTGTAATGTAGACAT
TCCTCTACGTTTGTTATTTGAAGCACCAACCATTCAGGAAATCTCTAATTATATAAAC
GGGGCAAAGAAAGAAAGCTATGTTGCCATTCAGCCTGTACCAGAACAAGAGTACTA
TCCTGTATCATCAGTTCAAAAAAGAATGTTTATTCTTAATGAATTTGATCGTTCAGGT
ACGGCCTATAATTTACCTGGTGTTATGTTTCTAGATGGAAAATTGAACTACCGACAA
TTGGAAGCAGCGGTAAAAAAATTAGTTGAGCGACATGAAGCGCTGCGTACTTCCTTT
CATTCAATTAATGGGGAACCAGTTCAGCGGGTGCATCAAAATGTAGAACTGCAGAT
TGCTTATTCAGAGTCAACGGAAGATCAGGTGGAGCGAATTATTGCGGAATTTATGCA
ACCATTTGCTCTTGAAGTTGCTCCGTTACTTCGTGTAGGTCTTGTTAAATTGGAGGCA
GAACGTCATCTATTTATAATGGATATGCATCATATCATCTCGGATGGGGTATCCATG
CAGATCATGATTCAAGAAATTGCTGATTTGTATAAAGAAAAGGAACTTCCTACGTTA
GGCATTCAATATAAAGACTTTACTGTTTGGCATAATCGCTTGCTTCAATCGGATGTTA
TTGAAAAACAAGAAGCTTACTGGCTGAACGTATTTGCAGAAGAGATTCCAGTATTG
AATCTACCGACCGATTACCCAAGACCAACCATTCAAAGCTTTGATGGTAAAAGATTT
ACATTCAGTACAGGAAAGCAGCTTATGGATGATTTATACAAGGTGGCAACAGAAAC
AGGAACAACACTATATATGGTTTTACTTGCTGCGTATAATGTTTTCTTATCGAAGTAT
TCCGGGCAAGATGACATCGTTGTAGGAACACCGATTGCTGGTAGGTCCCATGCTGAT
GTGGAAAATATGCTGGGGATGTTTGTAAATACATTAGCAATAAGAAGTCGTTTAAAT
AATGAGGATACTTTTAAAGATTTTTTAGCAAATGTAAAACAAACGGCTTTGCATGCC
TATGAAAATCCAGATTACCCATTTGATACGCTTGTCGAAAAGTTGGGTATACAGAGA
GATTTAAGTAGAAATCCATTATTTGATACGATGTTTGTTTTGCAAAATACGGATAGA
AAGTCTTTTGAGGTTGAACAGATAACGATTACACCATATGTTCCAAATAGCAGACAT
TCTAAATTTGATCTTACATTAGAGGTTAGCGAAGAACAAAATGAGATTTTATTATGC
CTAGAATATTGCACTAAATTATTTACGGATAAAACAGTTGAAAGAATGGCTGGTCAT
TTTTTACAGATCTTGCATGCAATTGTTGGGAACCCAACGATTATAATATCAGAAATC
GAGATATTGTCTGAAGAAGAAAAACAACATATTTTATTCGAGTTCAACGATACGAA
AACCACATATCCACATATGCAAACAATTCAAGGATTATTTGAGGAACAGGTGGAGA
AAACGCCCGACCATGTTGCAGTTGGATGGAAAGACCAAACATTAACGTATCGGGAA
CTTAACGAAAGAGCGAATCAGGTCGCAAGAGTCTTACGGCAAAAAGGAGTCCAACC
CGATAATATCGTGGGATTGCTGGTTGAGCGTTCACCTGAAATGCTCGTGGGTATCAT
GGGAATTCTTAAAGCAGGGGGAGCTTATTTACCTCTTGATCCGGAGTACCCAGCGGA
TAGAATTTCGTACATGATACAAGATTGTGGTGTACGCATTATGCTTACCCAACAGCA
TCTTTTATCTTTAGTACATGATGAATTTGATTGTGTTATTTTGGATGAGGACAGTTTG
TACAAGGGGGATTCTTCCAATTTGGCTCCGGTTAACCAGGCCGGGGATTTAGCCTAC
ATCATGTACACTTCTGGTTCTACAGGAAAGCCTAAAGGTGTTATGGTAGAACATCGA
AATGTGATTCGCCTTGTGAAAAATACAAATTATGTTCAGGTCCGCGAGGACGATCGT
ATAATACAGACCGGAGCAATTGGATTCGATGCACTGACATTTGAAGTTTTTGGCTCA
TTGCTGCATGGAGCTGAATTGTATCCTGTTACTAAAGACGTGCTATTAGATGCAGAG
AAACTACACAAATTTTTACAAGCGAATCAAATTACGATTATGTGGTTAACTTCTCCG
TTATTTAACCAATTGTCACAAGGAACCGAAGAGATGTTTGCTGGCCTTCGCTCCCTA

ATTGTAGGTGGAGATGCCTTGTCTCCGAAACACATCAATAATGTAAAGCGAAAATG
CCCTAATCTGACTATGTGGAACGGTTACGGCCCAACAGAAAACACCACTTTTTCTAC
ATGCTTTCTTATTGATAAAGAATATGATGACAATATTCCGATAGGGAAGGCCATTAG
TAATTCAACAGTGTATATCATGGACCGGTATGGCCAGCTTCAGCCGGTGGGTGTACC
AGGAGAATTATGTGTAGGAGGGGATGGGGTTGCCAGGGGATATATGAATCAGCCTG
CATTAACAGAAGAGAAGTTTGTCCCAAATCCATTCGCTCCTGGTGAGAGAATGTATC
GCACGGGGGATTTGGCAAGATGGTTGCCTGATGGAACAATTGAGTATTTAGGTCGTA
TTGATCAGCAAGTGAAAATCAGGGGCTACCGTATTGAACCGGGAGAGATTGAAACG
CTTCTTGTGAAGCACAAAAAAGTCAAAGAATCGGTAATCATGGTAGTAGAGGATAA
TAATGGACAAAAGGCTCTATGCGCTTATTACGTTCCGGAAGAAGAAGTAACGGTAT
CTGAACTGAGGGAATATATAGCTAAAGAGTTGCCTGTTTACATGGTTCCAGCCTATT
TTGTACAGATTGAACAAATGCCTCTTACACAGAACGGTAAAGTAAATCGAAGCGCG
TTACCAAAACCAGATGGTGAATTTGGTACAGCAACCGAATATGTAGCGCCTAGCAG
CGACATTGAAATGAAGCTGGCAGAGATTTGGCATAATGTGTTAGGGGTAAACAAAA
TCGGGGTACTGGATAACTTCTTTGAATTAGGTGGTCATTCATTAAGAGCTATGACAA
TGATTTCCCAGGTACATAAAGAGTTCGACGTTGAATTGCCATTAAAAGTGTTATTTG
AAACACCAACGATCTCTGCATTAGCTCAATACATTGCTGATGGAGAAAAAGGAATG
TACCTGGCCATTCAACCTGTTACCCCGCAGGATTACTATCCAGTATCATCTGCGCAA
AAGAGGATGTACATCCTTTATGAATTTGAAGGGGCTGGCATTACCTATAATGTACCT
AATGTAATGTTTATAGAAGGAAAGCTGGATTATCAGCGCTTTGAATACGCTATAAAA
AGTTTGGTAAATCGACATGAGGCGCTTCGAACGTCTTTCTATTCGCTTAATGGAGAA
CCAGTTCAGCGTGTACATCAAAATGTAGAGCTACAGATTGCTTATTCGGAGGCGAAA
GAAGATGAGATAGAGCAAATTGTAGAAAGCTTTGTTCAACCATTTGACCTTGAAATA
GCTCCGCTGCTTCGCGTAGGGCTTGTTAAATTGGCATCGGATCGCTATTTATTCCTAA
TGGATATGCATCATATTATCTCAGATGGTGTATCAATGCAAATTATAACAAAAGAAA
TTGCCGACTTATATAAAGGAAAAGAGCTTGCTGAACTGCATATTCAGTATAAAGATT
TTGCTGTATGGCAAAACGAATGGTTTCAATCTGACGCTCTTGAAAAACAGAAAACGT
ATTGGTTGAACACCTTTGCAGAGGATATTCCGGTTTTAAATTTGTCAACTGATTATCC
AAGACCGACAATTCAAAGTTTTGAAGGAGATATTGTCACGTTTAGTGCAGGGAAGC
AACTTGCGGAAGAATTGAAACGCCTGGCTGCAGAAACAGGGACGACTTTGTATATG
CTTCTGTTAGCGGCGTACAATGTACTTTTACACAAATACTCGGGACAGGAAGAAATT
GTAGTAGGAACGCCTATTGCCGGGCGATCTCACGCAGATGTGGAAAATATTGTTGG
GATGTTTGTCAATACGCTTGCATTGAAAAATACCCCTATAGCCGTACGCACCTTCCA
CGAATTCCTGTTGGAAGTAAAACAAAATGCTTTAGAAGCTTTTGAAAATCAAGACTA
TCCATTTGAAAATTTGATAGAGAAGCTGCAAGTGCGTCGCGACTTAAGTCGCAATCC
ATTATTTGATACAATGTTTAGCCTAAGCAATATTGACGAACAAGTAGAGATAGGGAT
TGAGGGATTGAACTTCAGCCCATATGAAATGCAGTATTGGATTGCAAAATTTGATAT
TTCATTCGATATTTTAGAAAAGCAAGATGACATTCAATTTTATTTTAACTATTGCACG
AATCTGTTTAAAAAAGAAACGATAGAACGATTAGCGACACACTTTATGCATATTTTA
CAGGAGATTGTTATTAATCCTGAGATTAAGTTATGTGAAATTAATATGCTGTCCGAA
GAAGAACAGCAGCGTGTCCTGTATGACTTTAATGGCACAGATGCAACCTACGCTAC
GAATAAAATATTCCATGAGTTATTTGAAGAACAGGTTGAAAAAACACCAGATCATA
TAGCGGTGATAGATGAAAGAGAAAAGCTTTCCTATCAGGAGCTTAATGCGAAAGCG
AATCAGCTGGCACGAGTGCTGCGCCAAAAAGGAGTACAGCCTAATAGCATGGTAGG
TATTATGGTAGATCGCTCACTCGACATGATTGTAGGAATGCTTGGGGTTTTAAAAGC
AGGAGGAGCATATGTGCCTATCGATATAGACTATCCTCAGGAACGGATTAGCTACAT

GATGGAAGATAGTGGTGCAGCGCTCTTGTTAACACAACAAAAGTTGACACAGCAAA
TTGCGTTTTCTGGTGACATTTTGTATCTTGACCAAGAAGAATGGCTTCATGAGGAAG
CTTCAAATTTAGAACCCATCGCTCGTCCGCAGGATATAGCCTATATCATTTACACTTC
TGGTACAACCGGAAAGCCAAAAGGTGTGATGATTGAGCATCAAAGCTATGTGAATG
TAGCAATGGCATGGAAAGATGCCTATCGGTTAGATACATTCCCGGTCCGTTTGCTTC
AGATGGCTAGCTTTGCCTTTGACGTATCTGCGGGTGATTTTGCCAGAGCACTACTTA
CAGGTGGGCAATTAATTGTATGTCCAAATGAAGTAAAGATGGACCCAGCTTCTTTAT
ATGCCATTATTAAGAAATATGACATTACTATTTTTGAAGCAACGCCTGCTCTAGTGA
TTCCATTGATGGAGTATATTTATGAACAGAAGCTGGATATTAGCCAGTTACAGATTC
TGATTGTCGGATCGGACAGTTGTTCGATGGAGGACTTTAAAACCTTGGTTTCCCGTTT
TGGTTCAACTATACGTATTGTGAATAGCTATGGAGTAACCGAAGCGTGCATTGATTC
TAGCTATTATGAACAACCGCTTTCTTCGTTACATGTAACAGGAACTGTACCGATTGG
AAAACCGTACGCTAACATGAAAATGTATATTATGAATCAATATTTGCAGATTCAGCC
TGTAGGTGTAATTGGAGAATTATGTATTGGAGGAGCCGGGGTTGCCCGTGGATATTT
AAATAGACCGGACTTAACAGCAGAAAAGTTTGTCCCTAATCCTTTTGTTCCAGGTGA
AAAGCTGTATCGAACAGGCGACTTGGCAAGATGGATGCCGGATGGGAATGTTGAGT
TTCTTGGTCGAAATGACCATCAGGTGAAAATCAGAGGGATTCGAATCGAGCTTGGA
GAAATCGAAGCACAACTGCGTAAACATGATAGCATAAAAGAAGCAACTGTGATCGC
AAGAGAAGATCACATGAAAGAGAAATATTTATGTGCGTATATGGTGACCGAAGGAG
AAGTAAATGTAGCTGAACTGCGTGCGTATCTAGCAAATGATCTGCCTGCGGCAATGA
TTCCGTCATATTTTGTATCGCTCGAAGCAATGCCACTTACTGCTAATGGAAAAATTG
ATAAGCGATCTTTACCAGAGCCCGATGGTTCCATATCGATAGGAACAGAATATGTAG
CTCCGCGTACCATGCTTGAGGGAAAACTAGAAGAGATATGGAAAGATGTATTGGGT
TTACAGCGTGTTGGCATTCACGATGACTTCTTTACAATAGGTGGCCATTCATTGAAG
GCTATGGCTGTTATTTCGCAAGTTCATAAAGAATGCCAGACTGAAGTTCCTCTGCGT
GTCTTATTTGAAACACCTACCATTCAAGGACTGGCTAAATATATAGAGGAAACGGAC
ACAGAGCAATATATGGCTATTCAGCCGGTTAGCGGACAGGACTATTATCCAGTATCA
TCAGCACAAAAGAGAATGTTTATTGTTAATCAATTTGATGGAGTAGGAATTAGCTAC
AATATGCCTTCCATCATGCTGATTGAAGGAAAACTTGAGCGAACACGCTTGGAATCA
GCATTTAAAAGATTGATAGAACGACATGAGAGCCTTCGAACATCTTTTGAAATAATA
AATGGTAAGCCTGTACAGAAGATTCATGAGGAAGTTGATTTCAATATGTCCTATCAG
GTGGCTTCTAATGAACAAGTAGAGAAGATGATCGATGAGTTCATTCAGCCTTTCGAT
TTAAGTGTTGCACCGCTGCTTCGTGTGGAACTTTTAAAATTGGAAGAGGACCGTCAT
GTGCTTATATTTGATATGCATCATATTATCTCAGATGGTATATCTTCCAATATTTTGA
TGAAAGAATTAGGAGAACTATATCAAGGTAATGCTTTACCAGAACTTCGTATTCAAT
ACAAGGATTTCGCTGTATGGCAAAATGAGTGGTTCCAGTCAGAAGCCTTTAAAAAG
CAAGAAGAATACTGGGTAAATGTTTTCGCAGATGAACGCCCGATTCTGGATATACCG
ACGGATTATCCAAGGCCGATGCAACAAAGCTTTGATGGTGCTCAACTTACATTTGGA
ACCGGAAAGCAGCTTATGGATGGGTTATACAGGGTAGCAACGGAAACGGGAACAAC
GCTTTATATGGTTTTGCTTGCGGCATATAATGTTCTTCTTTCCAAATATTCTGGTCAA
GAAGATATTATTGTAGGGACACCGATTGTGGGTAGATCCCATACTGACCTTGAGAAT
ATTGTCGGGATGTTTGTCAACACGTTAGCAATGAGAAATAAACCGGAAGGAGAAAA
GACGTTCAAAGCATTTGTATCAGAAATAAAGCAGAATGCACTAGCGGCTTTTGAGA
ATCAGGATTATCCATTTGAGGAGCTTATCGAAAAACTAGAGATACAAAGGGACTTA
AGCAGAAATCCATTATTTGATACGCTCTTTAGCCTTCAAAACATAGGTGAAGAATCA
TTTGAACTAGCCGAATTAACATGCAAACCTTTCGATTTGGTAAGCAAATTAGAGCAT

GCCAAGTTTGATCTGAGTCTTGTGGCAGTAGAAAAAGAGGAAGAAATTGCATTTGG
GCTTCAATACTGCACAAAACTGTATAAGGAAAAAACAGTTGAACAACTGGCTCAAC
ATTTTATTCAAATAGTAAAAGCAATTGTAGAAAATCCAGATGTCAAATTATCTGATA
TTGATATGTTATCTGAAGAAGAGAAGAAACAAATCATGCTTGAGTTCAATGATACG
AAAATACAATATCCGCAGAATCAAACAATACAGGAATTGTTTGAGGAGCAAGTGAA
GAAAACACCTGAACATATAGCAATCGTATGGGAAGGGCAAGCATTAACCTATCATG
AGCTAAATATAAAAGCTAATCAGTTAGCTCGTGTATTACGAGAAAAAGGGGTAACC
CCTAATCATCCTGTAGCGATTATGACGGAACGCTCATTAGAGATGATCGTAGGTATC
TTTAGTATTTTGAAAGCAGGAGGAGCATATGTTCCAATTGATCCAGCCTATCCACAA
GAACGTATTCAATACTTGCTTGAAGATAGCGGAGCGACGCTACTGCTTACTCAGTCA
CATGTATTAAATAAATTACCGGTCGATATCGAATGGTTGGATCTTACAGATGAACAA
AACTATGTAGAAGATGGTACCAATCTTCCATTTATGAATCAGTCAACAGATCTTGCC
TATATTATTTATACATCCGGTACAACAGGCAAGCCTAAAGGGGTTATGATTGAACAT
CAAAGCATCATCAACTGCCTGCAATGGCGGAAGGAAGAATACGAATTTGGACCAGG
GGATACGGCTCTACAAGTGTTTTCCTTTGCTTTTGATGGATTTGTAGCAAGTTTGTTT
GCTCCGATTCTTGCTGGTGCAACGTCTGTTCTCCCTAAGGAGGAAGAAGCAAAAGAT
CCAGTTGCATTGAAAAAACTGATCGCATCAGAAGAGATTACACATTACTACGGTGTG
CCTAGTTTGTTTAGTGCCATTCTTGATGTTTCTTCTAGTAAGGATTTGCAAAATTTAC
GCTGCGTCACTTTGGGAGGAGAGAAATTACCGGCTCAAATTGTTAAAAAAATCAAA
GAAAAAAATAAAGAAATTGAAGTCAACAACGAATATGGGCCTACTGAAAATAGTGT
AGTAACTACTATTATGCGCGATATACAGGTAGAACAAGAGATTACTATTGGTCGCCC
ATTATCTAACGTAGATGTATATATTGTCAATTGTAATCATCAATTACAACCAGTAGG
TGTAGTAGGGGAATTATGTATTGGTGGACAGGGACTTGCAAGAGGATATTTGAATA
AACCAGAGCTTACAGCAGATAAATTTGTTGTAAATCCATTCGTACCTGGTGAACGTA
TGTACAAAACCGGTGACCTTGCAAAATGGCGCTCAGATGGAATGATTGAATATGTG
GGCGTGTTGATGAACAAGTAAAAGTAAGAGGATATCGGATTGAGCTTGGTGAAAT
TGAATCAGCTATCCTAGAATACGAAAAAATTAAGGAAGCGGTAGTTATGGTTTCGG
AGCATACTGCATCTGAACAGATGTTATGTGCTTATATTGTAGGGGAAGAAGATGTAC
TGACTCTGGACTTAAGAAGCTATCTAGCAAAATTACTACCAAGTTATATGATTCCAA
ACTATTTTATCCAATTGGATAGTATTCCGCTTACACCAAACGGTAAAGTGGATCGTA
AAGCATTGCCTGAACCTCAAACCATTGGCTTAATGGCAAGGGAGTATGTTGCACCAA
GGAATGAAATCGAAGCACAGCTAGTACTCATTTGGCAAGAGGTATTAGGAATAGAA
CTGATCGGTATTACCGATAATTTCTTTGAATTAGGAGGGCATTCTTTAAAGGCAACG
CTTTTAGTTGCAAAAATTTACGAGTACATGCAAATAGAGATGCCATTAAATGTTGTG
TTTAAACATTCAACTATTATGAAAATAGCGGAATATATTACACATCAAGAATCAGAA
AATAATGTACATCAGCCTATTTTGGTAAATGTAGAAGCAGATAGAGAGGCGCTATCT
CTTAACGGCGAGAAGCAAAGAAAAAATATAGAGCTACCTATTCTGCTAAACGAAGA
AACAGATCGAAACGTATTCTGCTTCGCGCCCATTGGTGCACAAGGTGTTTTTTATAA
AAAGCTTGCTGAACAAATCCCTACTGCATCCTTGTATGGCTTTGACTTCATTGAAGA
TGATGATCGAATTCAGCAATATATTGAATCGATGATTCAAACTCAGTCAGACGGACA
ATATGTGCTAATTGGTTATTCTTCAGGAGGGAACCTGGCTTTTGAAGTAGCAAAAGA
AATGGAAAGGCAAGGATATAGTGTATCTGATTTGGTCTTGTTCGATGTTTACTGGAA
GGGAAAAGTATTCGAGCAAACAAAAGAAGAAGAAGAAAACATAAAAATAATA
ATGGAAGAATTAAGGGAAAATCCAGGAATGTTCAATATGACACGAGAGGATTTTGA
ACTGTATTTTGCGAATGAATTTGTGAAACAAAGTTTCACACGGAAAATGCGCAAATA
CATGAGTTTTTATACGCAGTTAGTTAATTATGGGGAAGTAGAAGCTACAATTCACCT

TATACAAGCAGAATTTGAGGAAGAAAAAATTGACGAAAACGAAAAAGCCGACGAA
GAAGAAAAAACATATCTAGAGGAAAAATGGAATGAAAAAGCATGGAACAAAGCAG
CAAAAAGATTTGTAAAATATAACGGATATGGCGCTCATTCTAACATGCTAGGAGGT
GATGGTTTAGAGAGAAATTCCTCTATCCTTAAACAGATACTACAAGGGACATTTGTA
GTAAAATAAAAGAAGAAGTGTGAAAAAGCGCAGCTGAAATAGCTGCGCTTTTTTGT
GTCATAA
